# Upregulation of cholinergic modulators Lypd6 and Lypd6b associated with autism drives anxiety and cognitive decline

**DOI:** 10.1038/s41420-024-02211-z

**Published:** 2024-10-21

**Authors:** Aizek B. Isaev, Maxim L. Bychkov, Dmitrii S. Kulbatskii, Alexander A. Andreev-Andrievskiy, Mikhail A. Mashkin, Mikhail A. Shulepko, Olga V. Shlepova, Eugene V. Loktyushov, Alexander V. Latanov, Mikhail P. Kirpichnikov, Ekaterina N. Lyukmanova

**Affiliations:** 1 Shemyakin-Ovchinnikov Institute of Bioorganic Chemistry, Russian Academy of Sciences, Moscow, Russia; 2 Moscow Center for Advanced Studies, Moscow, Russia; 3 Interdisciplinary Scientific and Educational School of Moscow University «Molecular Technologies of the Living Systems and Synthetic Biology», Faculty of Biology, Lomonosov Moscow State University, Moscow, Russia; 4 Institute for Biomedical Problems of Russian Academy of Sciences, Moscow, Russia; 5 Shenzhen MSU-BIT University, Shenzhen, China; 6 Pushchino Scientific Center for Biological Research of the Russian Academy of Sciences, Institute for Biological Instrumentation, Pushchino, Russia

**Keywords:** Cognitive neuroscience, Molecular neuroscience

## Abstract

Intellectual disability and autistic features are associated with chromosome region 2q23.q23.2 duplication carrying *LYPD6* and *LYPD6B* genes. Here, we analyzed *LYPD6* and *LYPD6B* expression in patients with different neuropsychiatric disorders. Increased *LYPD6* and *LYPD6B* expression was revealed in autism and other disorders. To study possible consequences of Lypd6 and Lypd6b overexpression in the brain, we used a mouse model with intracerebroventricular delivery of recombinant analogs of these proteins. A two-week infusion evoked significant memory impairment and acute stress. Both modulators downregulated hippocampal and amygdala dendritic spine density. No changes in synaptic plasticity were observed. Intracerebroventricular administration by both proteins downregulated hippocampal expression of Lypd6, Lypd6b, and α7 nicotinic acetylcholine receptor (nAChR). Similar to Lypd6, Lypd6b targeted different nAChR subtypes in the brain with preferential inhibition of α7- and α4β2-nAChRs. Thus, increased Lypd6 and Lypd6b level in the brain are linked to cholinergic system depression, neuronal atrophy, memory decline, and anxiety.

## Introduction

Nicotinic acetylcholine receptors (nAChRs) are ligand-gated ion channels that regulate processes such as learning, memory, nociception and behavior [1]. Dysfunction of the brain cholinergic system is associated with neurological and psychiatric diseases such as anxiety, schizophrenia, depression, olfactory memory decline, Parkinson’s disease (PD), and Alzheimer’s disease (AD), among others [1–3]. Dysregulation of α7 and β2 nAChR expression is a hallmark of bipolar disorder [4, 5] and AD [1, 6], α4β2-nAChR is downregulated in depression and PD [1], α5 subunit containing nAChRs are involved in the development of anxiety [7], and α7 and β2 nAChRs mediate cognitive decline in schizophrenia and autism [8–10].

Some endogenous proteins from the Ly6/uPAR family [11] (Lynx1 [12], Lynx2 [13], PSCA [14], Lypd6 [15, 16], Lypd6b [15, 17], SLURP-1 [18], SLURP-2 [19]) target nAChRs. Lynx1 and PSCA expression is decreased and increased, respectively, in the cortex in AD [6, 20], SLURP-1 expression is decreased in malignant cells [21]. Autistic spectrum disorders and intellectual disabilities are often accompanied by deletion or amplification of the chromosome region 2q23.q23.2, carrying *LYPD6* and *LYPD6B* [22, 23]. However, the role of the proteins coded by these genes in normal and pathological processes, particularly in cognitive function, as well as their influence on nAChRs in the brain remains poorly understood.

Lypd6 and Lypd6b, which share 54% sequence homology, are membrane-tethered Ly6/uPAR proteins expressed in the brain and non-neuronal tissues [16, 24, 25]. Lypd6 colocalizes with α7 and α3 nAChR subunits in the cortical and hippocampal neurons, inhibits α7- and α3β4-nAChRs, and suppresses long-term potentiation (LTP) ex vivo [15]. Lypd6 regulates juvenile visual plasticity [26], anxiety, and nociception [27]. Besides nAChRs, Lypd6 also interacts with Frizzled8 and LRP6 receptors enhancing the Wnt-signaling during embryonic development [28]. The pharmacology and role of Lypd6b in cognitive function is unknown.

Here, to establish possible relationships between Lypd6 or Lypd6b overexpression in the brain and neurological disorders, we performed bioinformatic analysis of tissues of patients with different neuropsychiatric diseases from the Gene Expression Omnibus. Enhanced mRNA Lypd6 and Lypd6b expression in the brain was found in patients with autism and some other pathologies. We modeled elevated levels of Lypd6 and Lypd6b in the mouse brain and studied how it affects cognitive function. We showed that increased abundance of Lypd6 and Lypd6b in the brain was associated with memory decline, anxiety, atrophy of hippocampal and amygdala dendritic spines, and downregulation of the cholinergic system. These findings offer new insight into the physiological role of Lypd6 and Lypd6b in the development of neuropsychiatric disorders.

## Results

### *LYPD6* and *LYPD6B* mRNA expression is increased in autism and other neuropsychiatric diseases

**Fig. 1:**
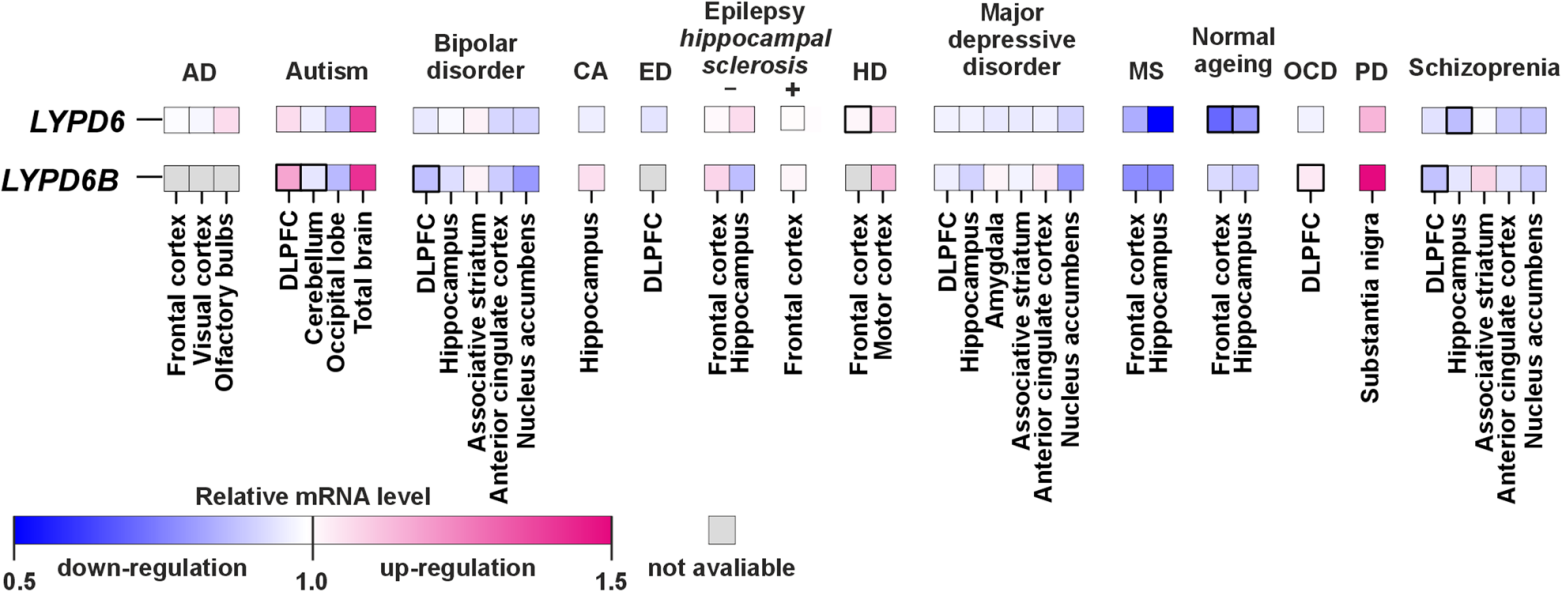
*LYPD6* and *LYPD6B* mRNA expression in the brain of patients with neurological and neuropsychiatric disorders. Data presented as relative mRNA level in diseased patients as compared to healthy donors. For the dataset accession numbers, number of patients and statistical details, see Supplementary Table 1. AD Alzheimer’s disease, CA chronic alcoholism, DLPFC dorsolateral prefrontal cortex, ED eating disorder, HD Huntington’s disease, MS multiple sclerosis, OCR Obsessive-compulsive disorder, PD Parkinson’s disease. For normal ageing, people younger 35 y.o. and older than 85 y.o. were compared. Boxes with the thick outlines represent significant difference between healthy donors and patients with neurological and neuropsychiatric disorders according to two-sided *t*-test. *p* values are in Supplementary Table 1.

Bioinformatic analysis revealed increased expression of *LYPD6* and *LYPD6B* mRNA expression in the brains of patients with different neurological and psychiatric disorders: autism, Huntington’s and Parkinson’s diseases, and in epilepsy (Fig. 1). A slight increase of *LYPD6* expression was observed also in the olfactory bulbs in AD. *LYPD6* was downregulated in schizophrenia and during normal aging. *LYPD6B* was upregulated in the cortex of patients with obsessive-compulsive disorder, slightly increased in the cortex and amygdala in major depressive disorder, in the striatum in bipolar disorder and schizophrenia, and in the hippocampus of patients with in chronic alcoholism. *LYPD6B* was downregulated in the cortex of patients with bipolar disorder and schizophrenia. Both genes were downregulated in multiple sclerosis (Fig. 1, Supplementary Table 1).

### Systematic therapy with Lypd6 and Lypd6b impairs cognitive function in mice

**Fig. 2:**
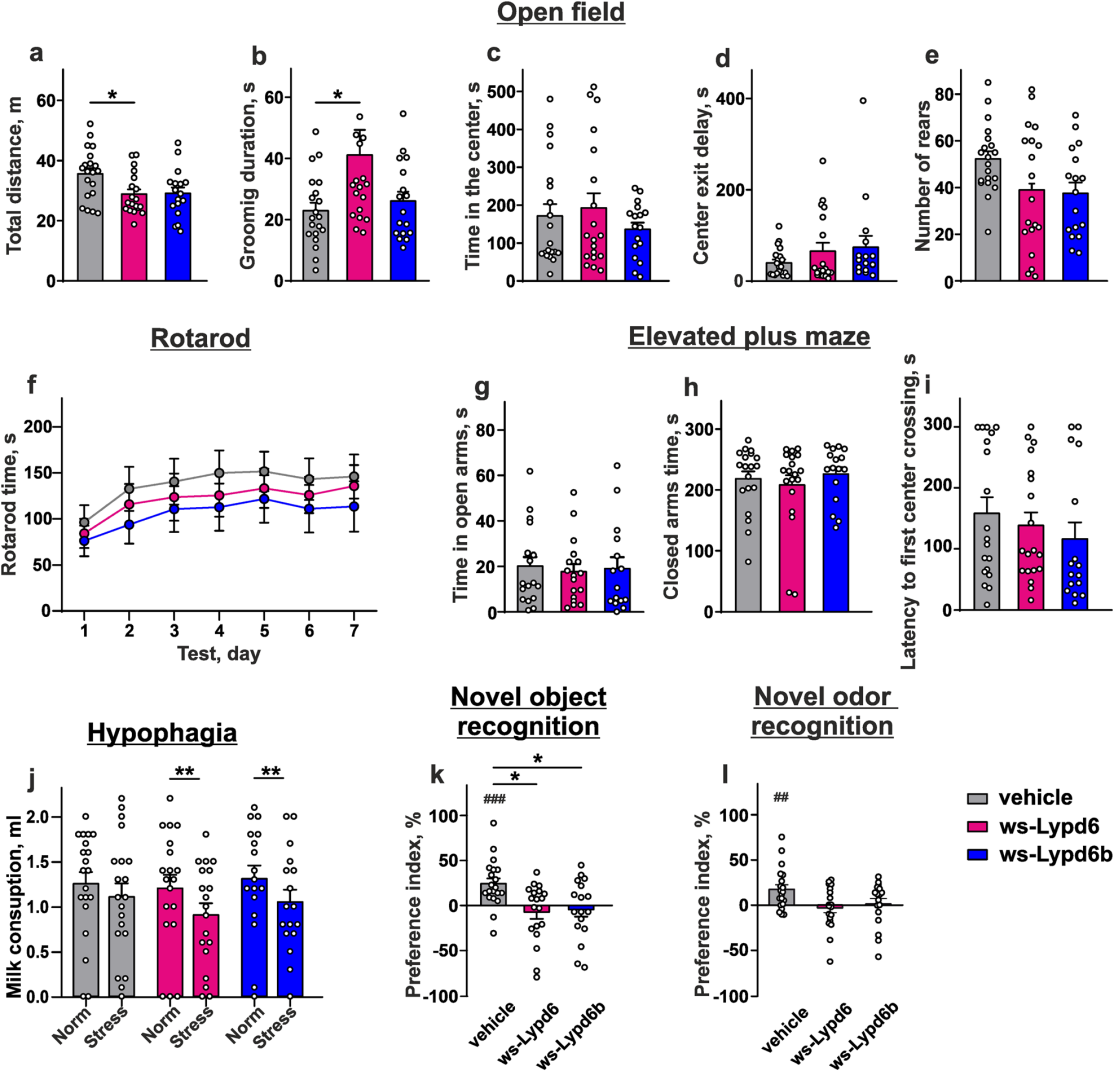
Working memory impairment and stress induction upon mice administration with ws-Lypd6 and ws-Lypd6b. **a**–**e** Open field test. Data presented as the value of an experimentally estimated parameter ± SEM (*n* = 17*–*20). *(*p* < 0.05) indicates significant difference from vehicle group according to Kruskal–Wallis test followed by *post hoc* Dunn’s test. **f** Rotarod test over the 7 consecutive days expressed as retention time on the accelerating rotarod roller (*n* = 17–19). No difference between the groups according to two-way ANOVA followed by *post hoc* Dunnet test. **g**–**i** Elevated plus maze. Data presented as the value of an experimentally estimated parameter ± SEM (*n* = 15–19). No difference between groups according to Kruskal–Wallis test followed by *post hoc* Dunn’s test. **j** Acute stress hypophagia test. Consumption of sweetened milk under comfortable conditions and upon acute stress in a novel environment. Data presented as sweetened milk consumption (ml) ± SEM (*n* = 17–20). *(*p* < 0.05) and **(*p* < 0.01) indicate significant difference between data groups according to two-way ANOVA with *post hoc* Sidak test. **k**, **l** Novel object and novel odor recognition tests. Data presented as preference index ± SEM (*n* = 17–20). ^##^(*p* < 0.01) and ^###^(*p* < 0.001) indicate significant difference in preference of novel object/novel odor over a familiar one according to one-sample Wilcoxon-test. *(*p* < 0.05) indicates significant difference between data groups according to Kruskal–Wallis test followed by *post hoc* Dunn’s test.

To model the upregulation of Lypd6 and Lypd6b in the brain during neurological diseases, we used a mouse model with intracerebroventricular delivery of recombinant water-soluble domains of Lypd6 and Lypd6b (ws-Lypd6 and ws-Lypd6b) into the brain. This approach overcomes the limitations of protein overexpression in defined brain regions only. To study the effects of wsLypd6 and ws-Lypd6b therapies on cognitive function, we performed a battery of behavioral tests on three groups of mice, receiving for two weeks, ws-Lypd6, ws-Lypd6b, or vehicle (control group). In the open field test, mice treated with ws-Lypd6 traveled shorter distances and groomed for longer periods compared to vehicle-treated mice (Fig. 2a, b). Both proteins did not alter the time spent in the arena center, latency to enter the center, and rearing (Fig. 2c–e). Similarly, the rotarod test did not show any changes in the locomotor performance of ws-Lypd6 and ws-Lypd6b treated mice in comparison to control ones (Fig. 2f).

Cognitive abilities can be impaired by anxiety and stress [29, 30]. The study of general anxiety in the elevated plus maze test revealed no differences in behavior between the experimental groups (Fig. 2g–i). However, when we investigated acute anxiety in the stress-induced hypophagia test by comparing sweet milk consumption of mice in their home cages and the unknown male’s cage, a significant reduction of milk consumption in the alien’s cage was observed for mice treated with the modulators (Fig. 2j).

Learning and memory were evaluated in the novel object recognition test. In this test, mice treated with vehicle expectedly spent more time investigating a novel object in comparison to a familiar one examined on the habituation day (Fig. 2k). In contrast, ws-Lypd6 and ws-Lypd6b treated mice explored both novel and old objects for the same time without differences in the object preference (Fig. 2k).

Neurodegeneration is often associated with declined olfactory memory [31]. The novel odour recognition test revealed that vehicle-treated mice spent more time investigating a novel odor than the familiar odor examined the day before. Contrarily, ws-Lypd6 and ws-Lypd6b treated mice demonstrated no bias towards a novel odor over the familiar one (Fig. 2l) indicating a deficiency in olfactory memory. Thus, an increase of Lypd6 and Lypd6b levels in the brain leads to stress induction and memory impairment.

### Ws-Lypd6 and ws-Lypd6b therapy leads to decreased density of stub and mushroom dendritic spines

**Fig. 3:**
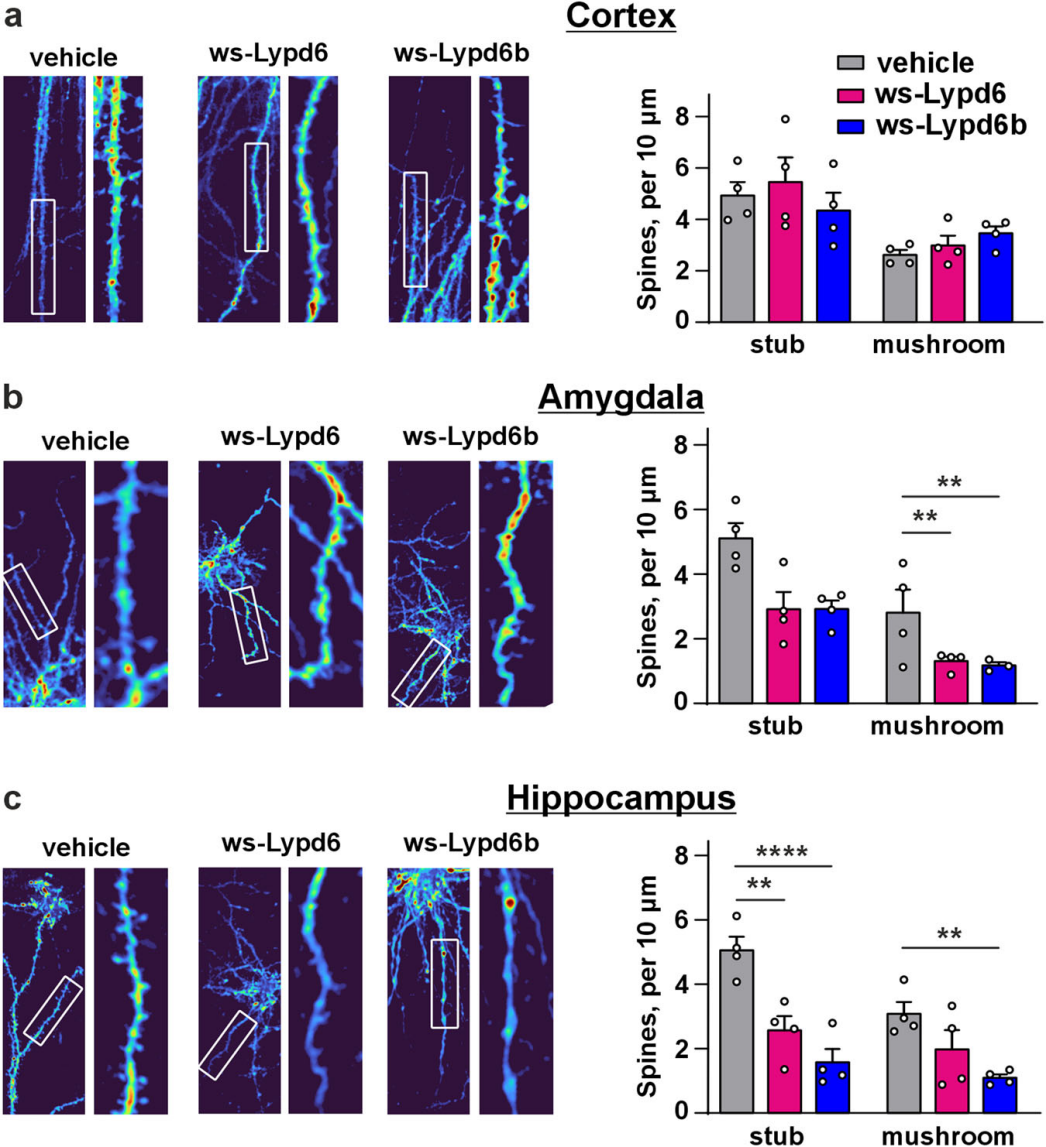
Decreased dendritic spine density in the amygdala and hippocampus following ws-Lypd6 and ws-Lypd6b administration in mice. **a**–**c** Left panels. Representative images of dendrites with spines of neurons from the cortex, amygdala, and hippocampus of experimental mice. **a**–**c** Right panels. Number of stub and mushroom-shaped dendritic spines in the cortex, amygdala, and hippocampus of treated mice. Data presented as a number of dendritic spines of different types (per 10 µm) ± SEM (*n* = 3*–*4). **(*p* < 0.01) and ****(*p* < 0.0001) indicate significant difference from vehicle group according to two-way ANOVA test following *post hoc* Dunnet test.

Stress-related memory impairment is linked with dendritic spines dysfunction [32–35]. We evaluated the influence of ws-Lypd6 and ws-Lypd6b administration on the number and morphology of dendritic spines in the cortex, hippocampus, and amygdala. Both modulators did not affect the density of dendritic spines in the frontal cortex of mice, but significantly reduced the number of mushroom spines in the amygdala and stub and mushroom-shaped spines in the hippocampus (Fig. 3). However, the ws-Lypd6 effect in the hippocampus did not reach the level of significance (*p* = 0.18, Fig. 3c).

### Ws-Lypd6 and ws-Lypd6b do not affect synaptic plasticity

**Fig. 4:**
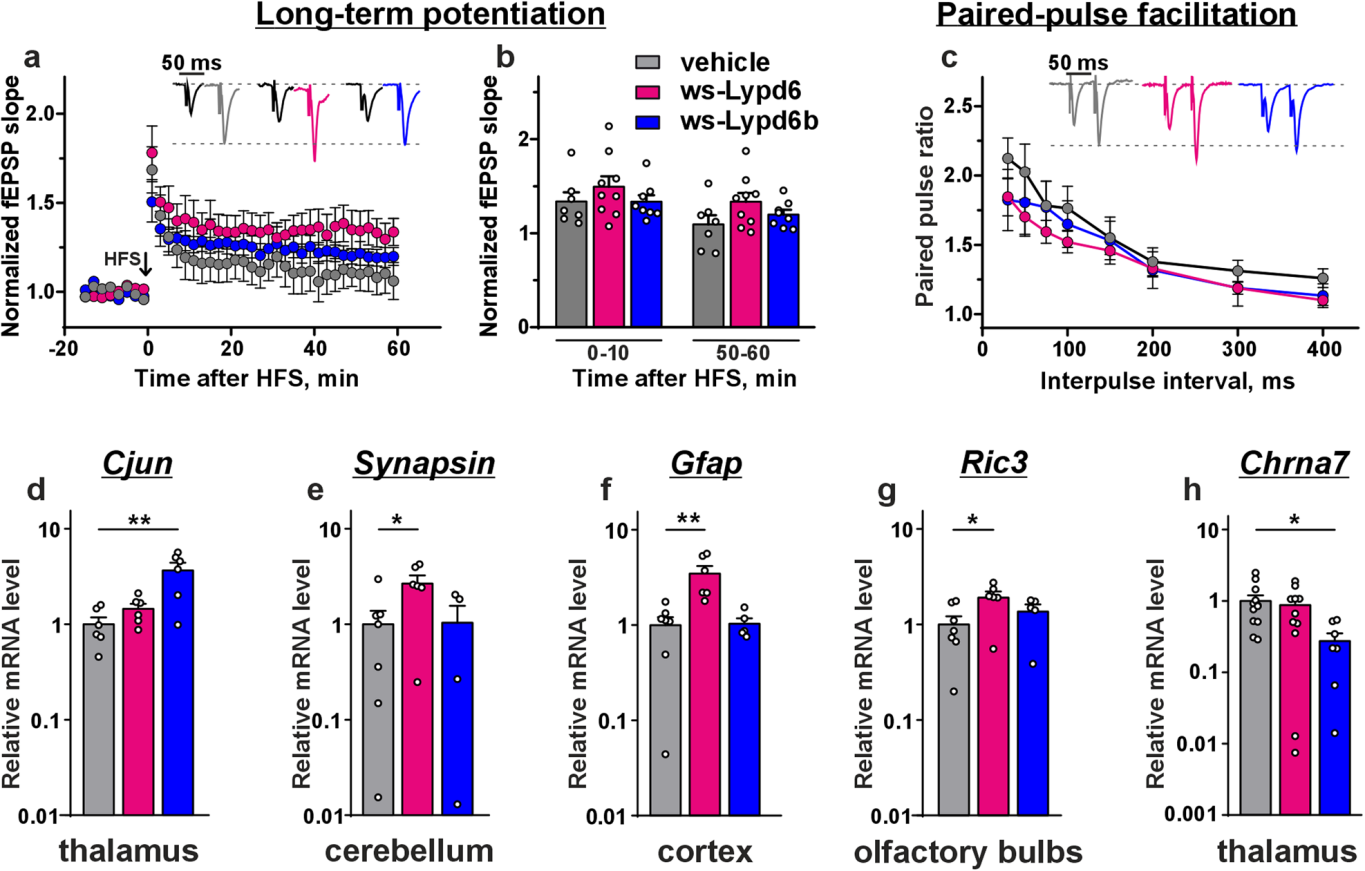
Ws-Lypd6 and ws-Lypd6b administration does not influence hippocampal synaptic plasticity. **a** Averaged normalized field excitatory postsynaptic potentials (fEPSPs) in hippocampal slices after drug administration were recorded for 1 h (*n*_ws-Lypd6_ = 9, *n*_ws-Lypd6b_ = 8, *n*_vehicle_ = 7) after HFS. Representative traces are shown above: black – baseline, colored – post-tetanic recording. **b** Normalized fEPSP slopes averaged over 0–10 and 50–60 min after HFS in the hippocampal slices. **c** PPF ratio upon stabilization of baseline fEPSP responses. Representative fEPSP traces for 50 ms interpulse interval stimulus are shown above. No difference between groups according to two-way ANOVA followed by *post hoc* Dunnett’s test (**a**, **c**) or one-way ANOVA followed by *post hoc* Dunnet test (**b**). **d**–**h** genes and brain regions that showed statistically significant differences in expression compared to the control group. Data are presented as the ratio of gene expression level ± SEM (*n* = 4–12). *(*p* < 0.05) and **(*p* < 0.01) indicate difference from control according to one-way ANOVA test followed by *post hoc* Dunnett’s test.

Changes in the density of hippocampal dendritic spines are usually accompanied by changes in LTP (an accepted model of synaptic plasticity [36]). However, no changes were observed in LTP following HFS or in paired-pulse facilitation ratio (PPF) in the hippocampus upon ws-Lypd6 and ws-Lypd6b therapy (Fig. 4a–c). We also studied mRNA expression of genes coding plasticity-related factors of plasticity in different brain regions: synaptic markers (Psd-95, Synapsin I, Synaptophysin, α-Synuclein), transcription factors (C-Fos, C-Jun), cytoskeletal filaments (GFAP, tubulin, actinin), Ly6/uPAR proteins (Lypd6, Lypd6b, Lynx1), α7nAChR and its chaperones NACHO and Ric3, and Lypd6’s target LRP6. Ws-Lypd6 increased gene expression for Synapsin I in the cerebellum, for filament Gfap in the cortex, and for Ric3 in the olfactory bulbs (Fig. 4d–g). For ws-Lypd6b, upregulation of mRNA coding transcription factor C-Jun and downregulation of mRNA coding α7 nAChR subunit were detected in the thalamus only (Fig. 4d, h). No changes in the expression of other studied genes were observed upon ws-Lypd6 or ws-Lypd6b administration (Supplementary Figs. 2 and 3).

### Ws-Lypd6 and ws-Lypd6b therapy changes expression of nAChRs, Lypd6, and Lypd6b in the cortex and hippocampus

**Fig. 5:**
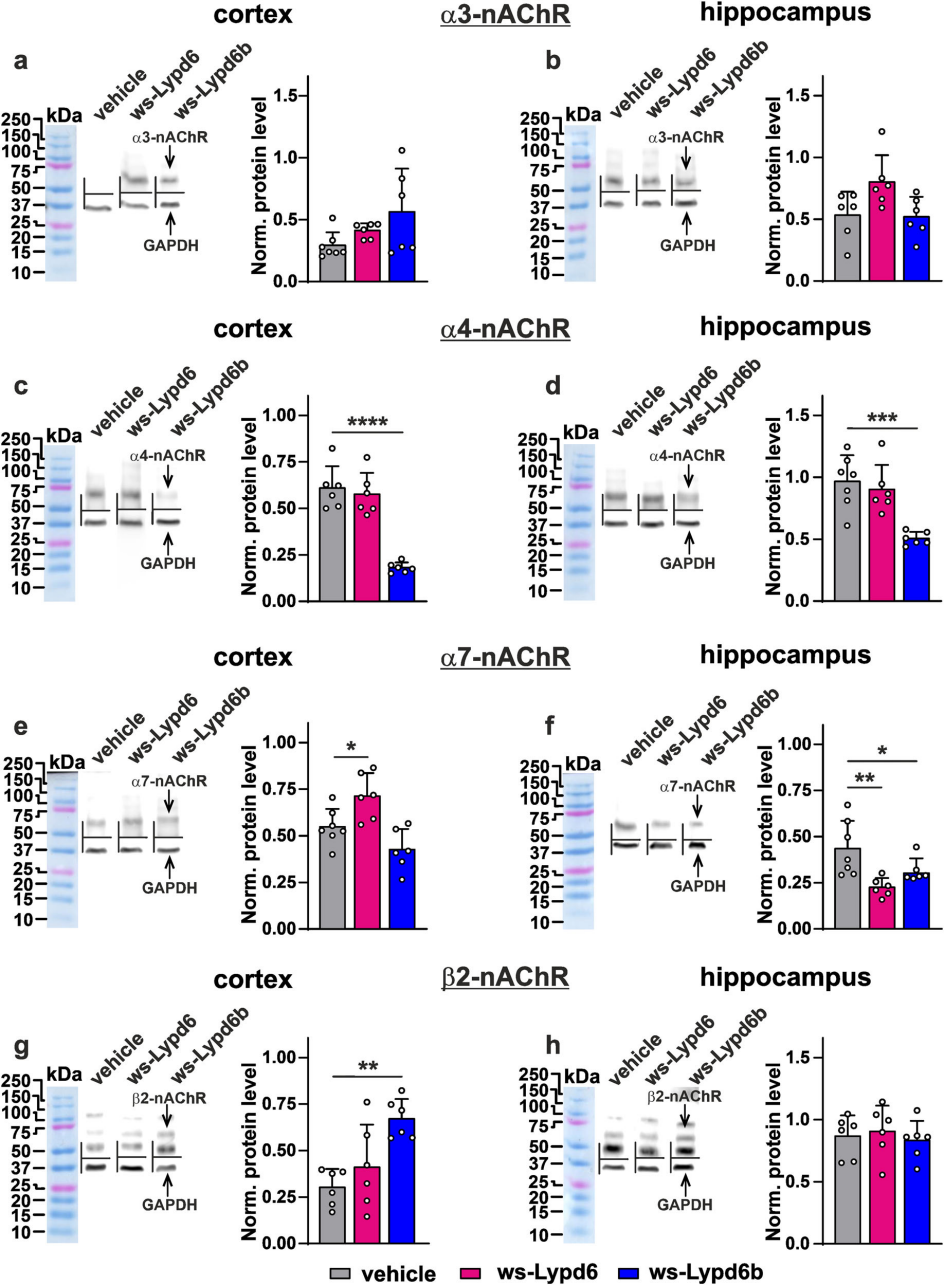
Changes in expression of α3-, α4-, α7-, and β2-nAChR subunits in the cortex and hippocampus upon ws-Lypd6 and ws-Lypd6b administration. **a**–**h** Left panels. Representative western blot bands for analysis of expression of α3-, α4-, α7-, and β2- nAChR subunits in the cortex (**a**, **c**, **e**, **g**) and hippocampus (**b**, **d**, **f**, **h**). **a**–**h** Right panels. Quantification of the α3-, α4-, α7-, and β2- nAChR subunits expression level normalized to GAPDH expression level. Data presented as normalized protein band intensity ± SEM (*n* = 6–7). *(*p* < 0.05), **(*p* < 0.01), and ****(*p* < 0.0001) indicate significant difference from vehicle group according to two-way ANOVA following *post hoc* Dunnett’s test. Whole western blotting membranes are in Supplementary Figs. 4 and 5.

To find an explanation for the behavioral effects and decreased dendritic spine density upon modulators administration, we analyzed the expression of α3, α4, α7, and β2 nAChR subunits and of endogenous modulators Lypd6 and Lypd6b in the cortex and hippocampus by immunostaining. Both neuromodulators downregulated hippocampal expression of the α7 subunit, and only ws-Lypd6b decreased expression of the α4 subunit in the hippocampus (Fig. 5d, f). In the cortex, wsLypd6b but not ws-Lypd6 decreased α4 subunit expression, ws-Lypd6 stimulated α7 subunit expression, and ws-Lypd6b increased β2 subunit expression (Fig. 5c, e, g). No effects on α3 subunit expression in the cortex and hippocampus, as well as on β2 subunit expression in the hippocampus were observed (Fig. 5a, b, h).

**Fig. 6:**
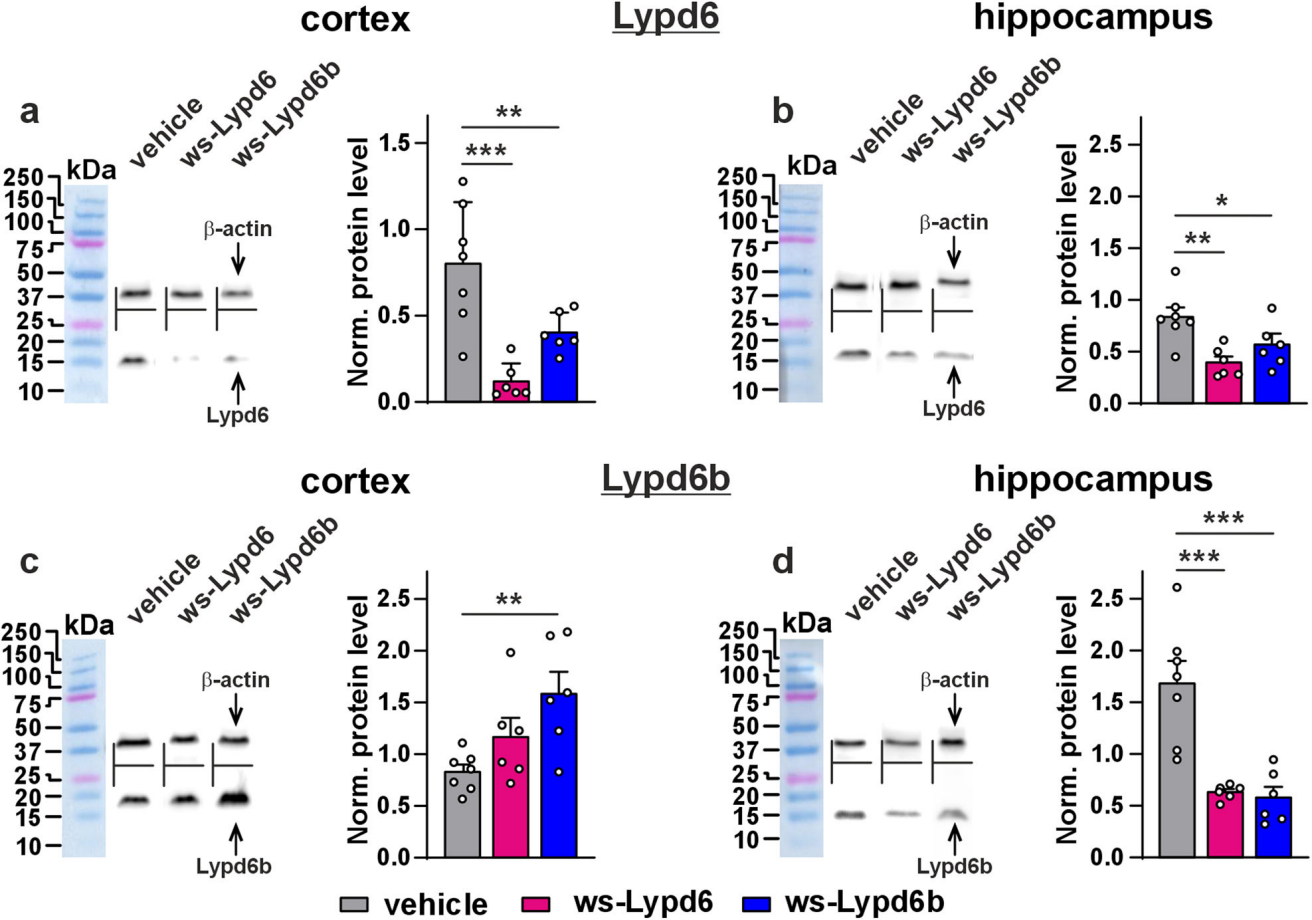
Changes in expression of endogenous Lypd6 and Lypd6b in the cortex and hippocampus upon ws-Lypd6 and ws-Lypd6b administration. **a**–**d** Left panels. Representative western blot bands for analysis of expression of endogenous Lypd6 and Lypd6b in the cortex (**a**, **c**) and hippocampus (**b**, **d**). **a**–**d** Right panels. Quantification of the Lypd6, Lypd6b expression level normalized to β-actin expression level. Data presented as normalized protein band intensity ± SEM (*n* = 6–7). *(*p* < 0.05), **(*p* < 0.01), and ****(*p* < 0.0001) indicate significant difference from vehicle group according to two-way ANOVA following *post hoc* Dunnett’s test. Whole western blotting membranes are in Supplementary Fig. 6.

Both neuromodulators downregulated endogenous Lypd6 expression in the cortex and hippocampus (Fig. 6a, b). Lypd6b expression was upregulated in the cortex upon ws-Lypd6b treatment and downregulated in the hippocampus upon both ws-Lypd6 and ws-Lypd6b treatment (Fig. 6c, d). Perhaps, decreased expression of endogenous Lypd6 and Lypd6b is the result of compensatory mechanisms activated in response to increased concentrations of the neuromodulators in the brain.

### Lypd6b targets α7-, α3β2-, and α4β2-nAChRs in the brain

**Fig. 7:**
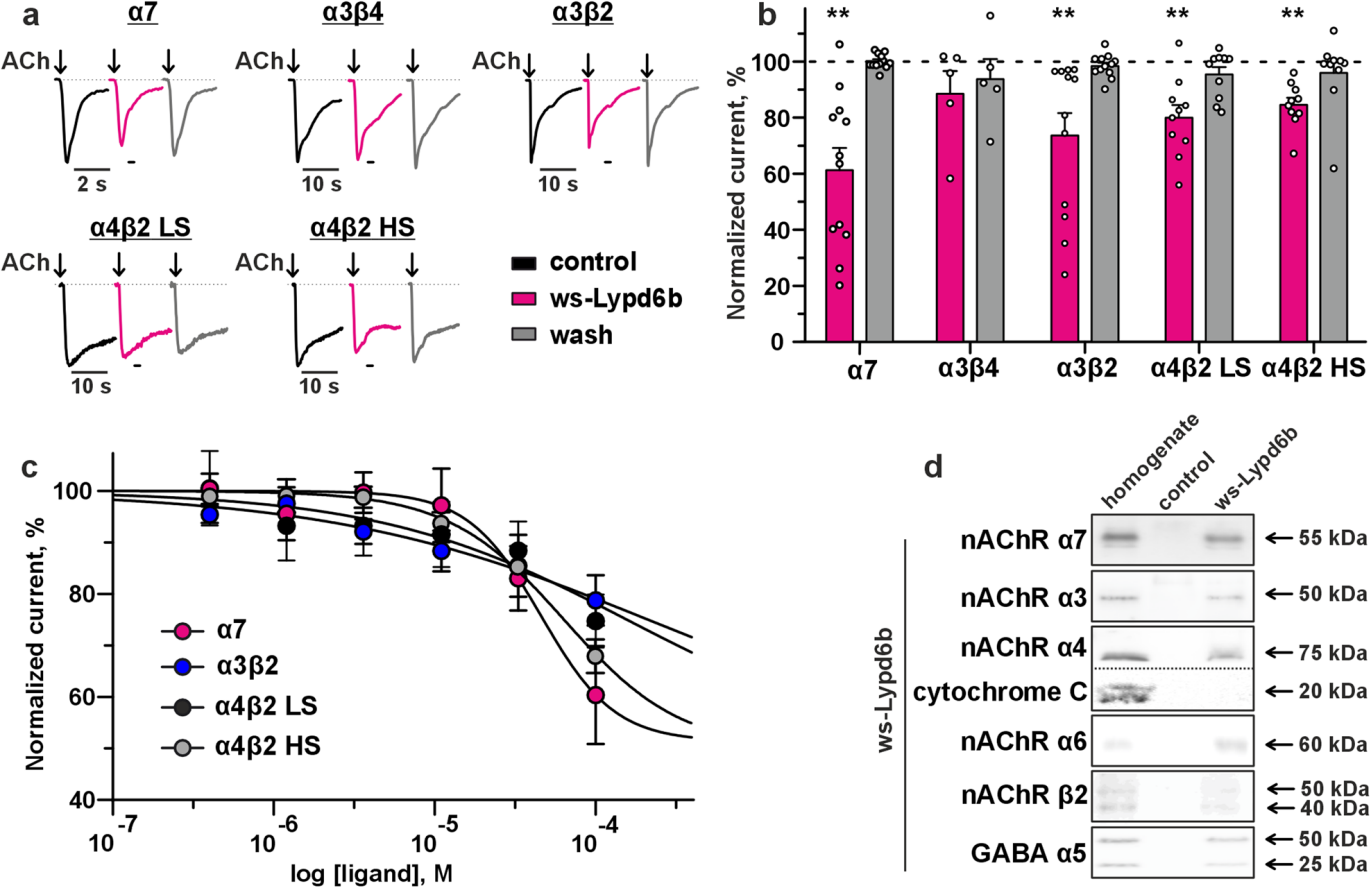
Ws-Lypd6b inhibits multiple nAChRs and binds subunits of different nicotinic and GABA_A_ receptors. **a** Normalized representative responses to 100 ms pulses of ACh (100 µM for α7, α3β2, α3β4, α4β2 LS, 10 µM for α4β2 HS) recorded at different nAChRs expressed in *X. laevis* oocytes in presence or absence of 30 μM ws-Lypd6b. Pre-incubation time of oocytes with ws-Lypd6b was 20 s. **b** Effect of 30 μM ws-Lypd6b on the ACh-evoked current at different nAChRs. Data presented as normalized ACh-evoked currents in the presence or the absence of ws-Lypd6b ± SEM (*n* = 5–13 oocytes). **(*p* < 0.01) indicates significant difference from control (100%, dashed line) according to one-sample Wilcoxon test following *post hoc* Holm-Sidak test. **c**. Dose-response curves for inhibition of ACh-evoked currents at different nAChRs by ws-Lypd6b. Data normalized to peak current amplitude recorded without ws-Lypd6b (100%), presented as mean ± SEM (*n* = 5–10 oocytes, each of them was treated by 0.5–100 µM ws-Lypd6b) and fit Hill’s equation. Please note that the difference in data between **b** and **c** is because experiments were carried out independently. **d** Analysis of the receptor subunits extracted by ws-Lypd6b from the total brain homogenate (*n* = 3–4). Empty NHS-Sepharose blocked with 500 mM ethanolamine was used as control. Whole western-blotting membranes are in Supplementary Fig. 7.

We previously found that ws-Lypd6 inhibits α7- and α3β4-nAChRs expressed in *Xenopus* oocytes and α7-nAChR in the brain [15]. Here, we studied the pharmacology of ws-Lypd6b using *Xenopus* *laevis* oocytes expressing the most abundant nAChR subtypes in the brain: α7-, α4β2-, α3β4-, and α3β2. In the presence of ACh, ws-Lypd6b demonstrated a reversible inhibition of α7-, α3β2-, and both isoforms of α4β2-nAChRs with low (stoichiometry (α4)_3_(β2)_2_, LS) and high (stoichiometry (α4)_2_(β2)_3_, HS) sensitivity to agonists. The most pronounced effect was observed at α7-nAChRs (Fig. 7a, b). No effect of ws-Lypd6b was observed at α3β4-nAChRs. This contradicts previous data about the inhibition of α3β4-nAChRs concatemers upon co-expression with membrane-tethered Lypd6b [17]. Perhaps, linking together of five nAChR subunits affects the ligand/receptor interaction. Notably, ws-Lypd6b application alone did not elicit currents at the receptors. Thus, Lypd6b is a reversible negative modulator of several types of nicotinic receptors.

The inhibition of ACh-evoked currents at α7-, α3β2-, and α4β2-nAChRs by ws-Lypd6b was concentration-dependent. However, a good fit to the Hill equation was obtained only for α7-nAChR and HS (α4)_2_(β2)_3_-nAChRs with the IC_50_ values of 46 ± 15 μM and 64 ± 15 μM, respectively, and maximal reduction of the current amplitude of ~60% and ~68%, respectively, relative to the control (Fig. 7c). For α3β2- and LS (α4)_3_(β2)_2_-nAChRs, IC_50_ values can be predicted in the range of ~200 μM, but the data for these receptors cannot be fitted well suggesting that the main targets of ws-Lypd6b are α7-nAChR and (α4)_2_(β2)_3_-nAChRs.

To prove the Lypd6b interaction with selected nAChR subtypes and to predict additional possible targets of this neuromodulator in the brain, we performed affinity extraction of α7, α3, α4, α6, and β2 nAChR subunits using ws-Lypd6b-conjugated Sepharose. Ws-Lypd6b extracted α7, α3, α4, α6, and β2 nAChR but not cytochrome C (used as a negative control) from mouse brain homogenate (Fig. 7d). Action on nicotinic receptors may be associated with the modulation of GABA_A_ receptors [37]. Indeed, ws-Lypd6b bound the α5 GABA_A_ receptor subunit (Fig. 7d), although further study of GABA_A_ receptors as possible targets of Lypd6 and Lypd6b is required. In general, our data points on low selectivity of ws-Lypd6b towards the receptors of CNS.

In order to establish the relevance of such interaction in the brain, we analyzed the localization of endogenous membrane-tethered Lypd6b and α7, α3, α4, α6, and β2 nAChR subunits in cortical and hippocampal neurons. We observed clear co-localization of Lypd6b with all these subunits in the neurons studied (Supplementary Fig. 8).

## Discussion

Bioinformatic analysis revealed increased or decreased *LYPD6* and *LYPD6B* expression in different neuropsychiatric disorders: autism, Parkinson’s and Huntington’s diseases, epilepsy, and others. However, in many cases, these differences did not reach statistical significance due to small sizes and heterogeneity of the groups (Fig. 1, Supplementary Table 1). Nevertheless, our results are supported by point clinical and scientific cases. For example, duplication of the chromosome region 2q23.q23.2 carrying *LYPD6* and *LYPD6B* genes was found in persons with autistic features [22, 23], and no changes in Lypd6 expression were observed in the cortex of patients with AD [6]. Thus, our data indicate that Lypd6 and Lypd6b may mediate at least some behavioral aspects of certain neurological disorders. The correlation between manifestations of neurological disorders and *LYPD6* and *LYPD6B* expression deserves further attention.

Mice receiving ws-Lypd6 exhibited impaired exploratory behavior, while both modulators caused acute context-dependent anxiety and impaired working and olfactory memory (Fig. 2). Decreased cognitive abilities and anxiety are characteristic of some (but not all) murine models of different neuropsychiatric diseases, such as autism, bipolar disorder, and schizophrenia [38–40]. Also, observed working memory deficiency resembles the behavior of newborn normal animals during a critical period of visual plasticity [41], when the expression of endogenous Lypd6 is highest in the brain [42]. In line with our data, *LYPD6* knockout in the brain resulted in a reduction of anxiety in mice [27].

To evaluate the structural and functional consequences of ws-Lypd6 and ws-Lypd6b administration, we analyzed the dendritic spine density and found its reduction in the hippocampus and amygdala (Fig. 3). Stress may be associated with suppressed dendritic spine density in the hippocampus [43] and amygdala [44, 45]. Thus, we can assume that impaired memory and anxiety of mice observed here may be driven at least partially by dendritic spine downregulation after ws-Lypd6 and ws-Lypd6b administration.

Memory and stress adaptation are associated with the dendritic spine density and turnover [46–49], while spine stabilization shares common mechanisms with regulation of synaptic plasticity [50]. No influence of ws-Lypd6 and ws-Lypd6b administration on LTP and expression of the factors of synaptic plasticity in the hippocampus [51–56] (Fig. 4, Supplementary Fig. 2) with simultaneous atrophy of dendritic spines (Fig. 3) points to the absence of a straightforward correlation between dendritic spine density and LTP examined before [57, 58]. The novel object recognition is associated with both structural and functional synaptic plasticity in the hippocampus [59]. Its impairment may be linked with the hippocampal dendritic spines downregulation [60]. Based on the data obtained, we suppose, that ws-Lypd6 and ws-Lypd6b affect only structural plasticity and drive compensatory adaptation, supporting synaptic transmission by unaffected dendritic spines. Also, the neuromodulators may target specialized subpopulations of the neurons, possibly from certain brain regions responsible for memory and anxiety. For example, endogenous Lypd6 and Lypd6b are expressed in GABAergic and glutamatergic interneurons of the visual cortex, respectively [25, 26]. Inhibition of some neurons may drive cognitive deficits with compensation of excitability of other neurons.

Formation, morphology, and turnover of dendritic spines can be controlled by nAChRs [61].

Activation of α7- and α4β2-nAChRs upregulates spine density [62, 63]. Ws-Lypd6 and ws-Lypd6b administration affected expression of endogenous α4, α7, and β2 nAChR subunits in the cortex and hippocampus (Fig. 5). Moreover, ws-Lypd6 inhibits α7-nAChRs [15], while ws-Lypd6b inhibits both α7- and α4β2-nAChRs (Fig. 7). Thus, memory decline and structural plasticity impairment observed here may be mediated both by downregulation of nAChRs expression and by its direct inhibition. α7-nAChR participates in the regulation of gene transcription in the brain [64], so its inhibition could affect the expression of numerous cognitive mediators including endogenous Lypd6 and Lypd6b (Fig. 6). Dopaminergic neurons expressing α4 containing nAChRs mediate anxiety relief in mice [65]. Thus, behavioral manifestations and structural atrophy of neuronal plasticity revealed here upon increased dosage of ws-Lypd6 and ws-Lypd6b in the brain can be mediated by changed function of different nAChRs.

Here, we studied ws-Lypd6b pharmacology in *Xenopus* oocytes and found inhibitory effects of the modulator on α7-, α3β2-, and α4β2-nAChRs (Fig. 7). The interaction of ws-Lypd6b with α3-, α4-, α6-, α7-, and β2-nAChR subunits extracted from the brain homogenate was confirmed (Fig. 7d). A similar recognition profile was reported previously for recombinant Lypd6 fused to glutathione S‑transferase, which extracted the α3, α4, α5, α6, α7, β2, and β4 nAChR subunits from the human brain homogenate [16]. Ws-Lypd6b binds the α5 subunit of the GABA_A_ receptor (Fig. 7d). Thus, the influence of systemic therapy by ws-Lypd6b on behavior and neuronal plasticity can be a result of interaction not only with nAChRs, but also with GABA_A_ and other receptors. Activation of α7nAChR with simultaneous inhibition of α5 containing GABA_A_ receptors by allosteric modulators stimulates LTP in the hippocampus [66]. Besides nAChRs, Lypd6 targets the Wnt receptor Frizzled8 and its co-receptor LRP6 [28], which are involved in dendritic spine formation [67]. We did not observe here an influence of ws-Lypd6 therapy on the expression of the *Lrp6* gene (Supplementary Fig. 3). However, we cannot exclude the interaction of recombinant modulators with these receptors in the brain. Thus, cognitive effects of ws-Lypd6 and ws-Lypd6b can be a sum of their interaction with different receptors. Perhaps, different targets of Lypd6 and Lypd6b expressed in different brain regions (Fig. 1) reflect the variative influence of these neuromodulators on cognitive function (Figs. 2 and 3).

A limitation of the present study is the approach used here to increase Lypd6 and Lypd6b concentration in the brain. We cannot control the distribution of neuromodulators in the brain upon intracerebroventricular administration, although the “targeted delivery” of the proteins into the preferred brain regions is expected due to the absence of GPI-anchor. On the other hand, gene-manipulating techniques based on viral transfection or gene knock-down also have their own limitations. Thus, gene knock-down affects protein expression throughout the organism, not only in the brain [16, 24, 28]. Viral transfection does not account for the concentration of the neuromodulators and the sensitivity of different brain parts to them. The alternative approach used here provides controlled administration of ws-Lypd6 and ws-Lypd6b into the brain in low doses (75 µM being accounted to the volume of the total brain) sufficient to affect nAChRs (Fig. 7).

**Fig. 8:**
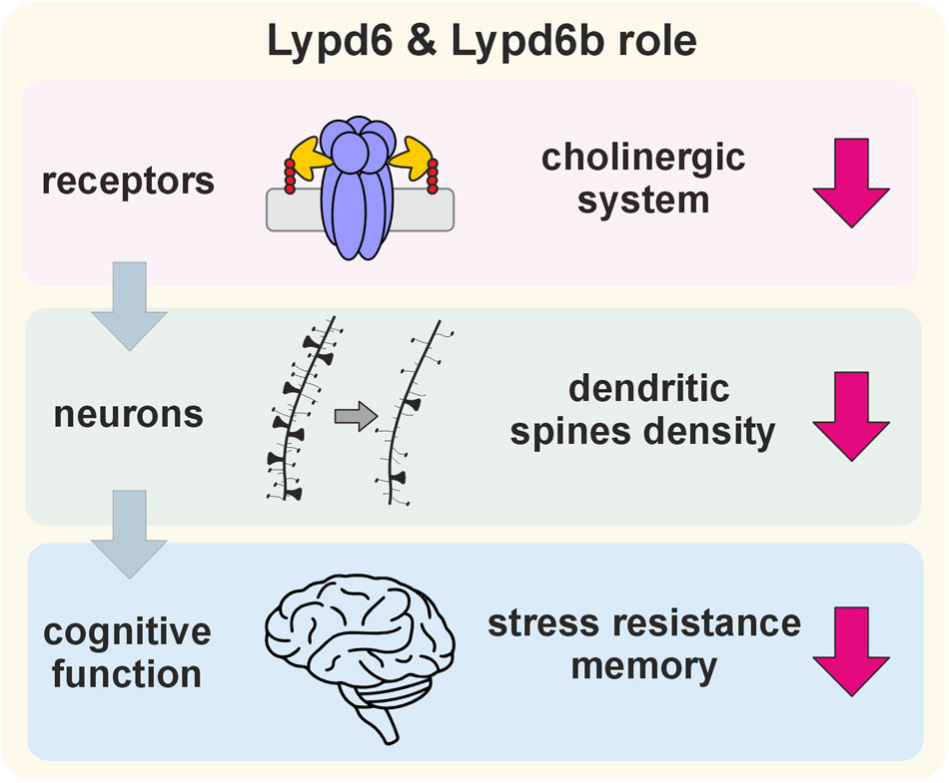
General proposed mechanism of Lypd6 and Lypd6b action in the CNS. Increased abundance of Lypd6 and Lypd6b in the brain impairs cognitive function at different physiological levels from neuronal receptors to neurons and behavior.

In conclusion, Lypd6 and Lypd6b are either upregulated or downregulated in different neuropsychiatric disorders. Increased expression of these neuromodulators in the brain may cause memory decline and anxiety associated with neuronal structural atrophy and suppressed cholinergic system but without impairment of synaptic plasticity. We propose that observed cognitive manifestations are mediated by inhibition of function and expression of different nAChRs (Fig. 8).

The details and participants of this chain of events have yet to be determined.

## Materials and methods

### Bioinformatic analysis

Data on *LYPD6* and *LYPD6B* expression in different brain regions of healthy donors and patients with neurological disorders were obtained from the Gene Expression Omnibus database and analyzed using Geo2R. Analysis details are given in Supplementary Table 1.

### Production of recombinant proteins

Ws-Lypd6 and ws-Lypd6b proteins were produced in *Escherichia coli* cells, refolded and characterized as described previously [68].

### Animals and drug administration

Male 16-week-old C57BL/6Ncrl mice (RRID: IMSR_CRL:027) were purchased from the Animal Breeding Facility BIBCh RAS and kept in individually ventilated cages; care and experiments were performed in accordance with the 86/609/EEC and 2010/63/EU directives on the protection of animals used for scientific purposes and were approved by IBCH RAS IACUC (protocol #312/2020 from 18 December 2020). Mice were randomized using software available online (randomization at (https://www.graphpad.com/quickcalcs/randomize1/). Ws-Lypd6 and ws-Lypd6b were infused intracerebroventricularly via stereotaxically implanted G30 cannulas connected to subcutaneously implanted osmotic pumps 2004D (Brain Infusion Kit 2, Alzet Cupertino, USA). Ws-Lypd6, wsLypd6b (5 mg/ml in PBS with 30% DMSO), or vehicle (PBS with 30% DMSO) were infused at a rate of ~6 μl/day (30 μg/day). Four-week stability of ws-Lypd6 and ws-Lypd6b samples at 37 °C was verified prior the animal study. Mice were sacrificed by cervical dislocation under isoflurane anesthesia.

### Behavioral test battery

Behavioral tests were performed following the 14th day of administration (Supplementary Fig. 1, Supplementary Table 2). For the open field test mice were placed in the center of a 60-cm round arena (400 l× white light, Open Science, Russia), and behavior was recorded for 10 min (20 fps). The elevated plus maze test was performed using a plastic arena (Open Science) with 40 cm opened (300 l×) and closed (40 l×) arms. Mice were placed into the center of the arena and behavior was recorded for 5 min (20 fps). The rotarod test (Neirobotics, Zelenograd, Russia) was performed over 7 days of the behavioral test battery. Each day, 5 trials (3 min with rod accelerated from 5 to 30 rpm) were performed. For the stress-induced hypophagia test, the change in the consumption of sweetened milk under stressful conditions compared with regular conditions was assessed. To habituate mice to test conditions, reconstituted sweetened condensed milk (Renna, Russia) was placed in the animal’s home cage. Milk consumption was measured over a 30-min observation period for 2 days. On the 3rd day, each mouse was placed into stressful conditions of an unfamiliar male’s cage, where milk consumption over 30 min was measure again. For the novel object recognition test, the animals were first habituated to the procedure by placing them twice a day for 3 min into a round dim plastic arena with two identical objects (plastic cubes or rubber dinosaurs of matching size). On the 4th day, after the last training session, the recognition of an unfamiliar object was tested. The final training and test sessions were recorded and analyzed. Object investigation was defined as presence of the mouse’s nose in a 2 cm zone around the object. Novel object preference index was calculated as a difference between the time for new and old objects investigation related to the total time of interaction with both objects. The olfactory memory (novel odor) test included 2-day training sessions, in which 2 strips of filter paper impregnated with 5 μl of urine from an unfamiliar same-sex mouse were placed in the mouse’s home cage at a distance of 5 cm from each other. On the 3rd day, one of the strips was impregnated by urine of an unfamiliar animal. Olfactory memory was assessed by the index of preference for a novel odor, calculated as the ratio of the time spent exploring the new and old odor to the total time of sniffing. Sessions were 5-min long. The videos were analyzed using EthoVision v. 15 (RRID: SCR_000441 Noldus, Netherlands). The investigator was blinded to the group allocation.

### Analysis of number and morphology of dendritic spines

Two hundred fifty micrometers thick transverse brain slices were initially fixed, stained by DiI in acetone (Sigma-Aldrich, St Louis, USA) for 16 h, fixed again, and DiI was allowed to diffuse for 140 h. Then, the slices were washed with PBS, embedded in Mowiol and observed using Carl Zeiss LSM710 (Carl Zeiss, Jena, Germany) confocal microscope under ×60 (1.4) oil immersion objective. Spines in 170 × 170 µm area (50 µm z-slice) were counted manually.

### Electrophysiological recordings in the hippocampal slices

Transverse hippocampal slices (350 µm thick) were incubated at 34 °C for 1 h in ACSF, pH 7.4 and bubbled with 95% O_2_ and 5% CO_2_. Field excitatory postsynaptic potentials (fEPSPs) were evoked by stimulating the Schaffer collaterals with bipolar electrode connected to Digitimer DS-3 stimulus isolator and recorded in hippocampal CA1 *str. radiatum* with 1–2 MΩ glass microelectrode connected to A-M Systems 1800 amplifier (Eindhoven, Netherlands). National Instruments PCI-6281 (Austin, USA) interface card and WinWCP 5.2.7 (Glasgow, UK) software were used for stimulus triggering and data recording. Stimulus intensity was calibrated to elicit 40% of maximal fEPSP amplitude. The stimulations were repeated at 20 s intervals. For PPF recording, paired pulse stimulations with 30–400 ms interpulse interval were used. Baseline was recorded for 20 min before LTP induction. LTP was induced by HSF stimulation: four 100-pulse trains at 100 Hz, 5 min apart. fEPSPs were then recorded for 60 min.

### Western blotting and immunostaining

Membrane fraction from the mouse cerebral cortex and hippocampus were isolated as in [69], blotted onto nitrocellulose membranes, blocked with 5% skim milk, and incubated overnight at 4 °C with the primary antibodies against Lypd6, Lypd6b, α3, α4, α7, and β2 nAChR subunits, washed, incubated with the HRP-conjugated secondary antibodies, washed, and the HRP signal was detected by the ECL substrate (Bio-Rad, Hercules, USA) using the ImageQuant LAS 500 chemidocumenter (GE Healthcare, Chicago, USA). The ECL signal was visualized in the chemiluminescent channel, and the protein marker was detected in the optical channel of the chemidocumenter. Intensity of the protein bands were normalized to the intensity of the bands of housekeeping proteins β-actin and GAPDH. For this, the same membranes were processed with anti-β-actin or anti-GAPDH antibodies. Ab cat# and dilutions are in Supplementary Table 3.

Protein expression was quantified using ImageQuant TL 8.2.0. software (GE Healthcare).

### Affinity extraction

Ws-Lypd6b was coupled to NHS-activated Sepharose 4 Fast Flow (17-0906-01, GE Healthcare) according to the manufacturer’s instructions. Sepharose blocked by 500 mM ethanolamine without any protein coupled was used as a negative control (empty resin). Affinity extraction of ws-Lypd6b targets from a membrane fraction of the total brain homogenate of adult mice was performed as in [18]. α3, α4, α6, α7, and β2 nAChR subunits, and GABAA α5 subunit were detected by western blotting and immunostaining. Cytochrome C was used as a negative control for experiments. Ab cat# and dilutions are given in Supplementary Table 3.

### Electrophysiological recordings in *X. laevis* oocytes

*X. laevis* oocytes (RRID: NXR_0.0080) were harvested and injected with mRNA coding nAChR subunits as in [15]. For heteromeric α3β2-nAChR, 1:1 ratio of α:β subunit mRNA was used. For expression of α4β2-nAChR with LS (α4)_3_(β2)_2_ and HS (α4)_2_(β2)_3_ stoichiometry, 10:1 and 1:10 ratios of α:β subunit mRNA were used, respectively. Two-electrode voltage-clamp recordings were done using the TEC-03X amplifier (NPI electronic GmbH, Tamm, Germany) at a holding potential of −50 mV, filtered and analyzed as in [15]. The currents were stimulated by application of 100 μM acetylcholine (10 μM for high-sensitive form of α4β2-nAChR) with subsequent 2–5 min washouts to prevent nAChR desensitization.

### Real-time PCR

Total RNA from the brain regions was isolated by the Aurum RNA Mini Isolation Kit (Bio-Rad, Hercules, USA). cDNA was synthesized by the Mint reverse transcriptase kit and oligodT primer (both - Evrogen, Moscow, Russia). qPCR was performed with ready-to-use SYBR Green HS mix (Evrogen) and the specific primers (Supplementary Table 4) using the Roche LightCycler 96 amplifier (Roche, Basel, Switzerland). The mRNA expression level was normalized to the *Atcb*, *Sdha*, and *RPL13a* housekeeping genes using the LightCycler SW software (Roche).

### Primary neuron culture and co-localization analysis

Neurons from the cortex and hippocampus were isolated as in [15]. For co-localization analysis, 14-day neurons were fixed, blocked with 1% BSA, incubated overnight with anti-Lypd6b antibody, washed, stained with Alexa647-conjugated anti-rabbit antibody, washed, blocked again, and then stained with antibodies against α3, α4, α7, and β2 nAChR subunits. Then, neurons were washed and incubated with TRITC-conjugated secondary antibodies. After washing, glasses were mounted in Mowiol-DABCO (Sigma-Aldrich) and observed under Carl Zeiss LSM710 confocal microscope (Carl Zeiss) under ×60 (1.4) oil immersion objective. Co-localization was analyzed using ImageJ.

Ab cat# and dilutions are given in Supplementary Table 3.

### Statistical analysis

Data are presented as mean ± SEM. The numbers of animals was determined according to previous studies [70, 71]. Outliers were removed by ROUT method (*Q* = 1%). The data were tested for normality (Shapiro-Wilk test, at *p* = 0.05). Sample numbers (*n*) and statistical tests are indicated in the figure legends. The differences in the data were considered statistically significant at *p* < 0.05. Analysis was performed using the GraphPad Prism 8.0 software (GraphPad Software, San Diego, USA).

## Supplementary information

Supplementary materials

Original WB images

## Data Availability

All data are available upon request. Original western blot membranes presented as supplementary file.
