# Supplementary material for: Upregulation of cholinergic modulators Lypd6 and Lypd6b associated with autism drives anxiety and cognitive decline
Source: Cell Death Discov. 2024 Oct 21;10:444. doi: 10.1038/s41420-024-02211-z (PMC11494011; doi:10.1038/s41420-024-02211-z)
Supplement: Supplementary file 1 — Supplementary materials [file 41420_2024_2211_MOESM1_ESM.docx]

**Up-regulation of cholinergic modulators Lypd6 and Lypd6b associated with autism drives anxiety and cognitive decline**

**Running title: Lypd6 and Lypd6b drive cognitive decline**

Aizek B. Isaev^2,3†^, Maxim L. Bychkov^2†^, Dmitrii S. Kulbatskii^2^, Alexander A. Andreev-

Andrievskiy^4,5^, Mikhail A. Mashkin^5^, Mikhail A. Shulepko^1^, Olga V. Shlepova^2,3^, Eugene V. Loktyushov^6^, Alexander V. Latanov^4^, Mikhail P. Kirpichnikov^2,4^, Ekaterina N. Lyukmanova^1,2,3,4^

^1^Shenzhen MSU-BIT University, 518172, Shenzhen, China

^2^Shemyakin-Ovchinnikov Institute of Bioorganic Chemistry, Russian Academy of Sciences, 119997, Moscow, Russia.

^3^Moscow Center for Advanced Studies, Moscow, Russia

^4^Interdisciplinary Scientific and Educational School of Moscow University «Molecular Technologies of the Living Systems and Synthetic Biology», Faculty of Biology, Lomonosov Moscow State University, 119234, Moscow, Russia.

^5^Institute for biomedical problems of Russian Academy of Sciences, 123007, Moscow, Russia

^6^Pushchino Scientific Center for Biological Research of the Russian Academy of Sciences, Institute for Biological Instrumentation, 142290, Pushchino, Russia *Correspondence: lyukmanova_ekaterina@smbu.edu.cn (E.N.L.)

† These authors contributed equally to this work

## Supplementary Table 1. *LYPD6* and *LYPD6B* expression in patients with neurological and neuropsychiatric disorders.

## Data on *LYPD6* and *LYPD6B* expression in different brain region of healthy donors and patients with neurological and neuropsychiatric disorders were taken from the Gene Expression Omnibus and analyzed using Geo2R. Fold change is the difference between expression level in diseased patients and healthy donors (control, the value for healthy donors is taken as “1”). “n/a”: the gene was not available in the dataset, LFC is Log2 expression change as compared to the expression level in healthy donors. *p* – *p*-value, t – size of the difference relative to the variation in data, B – log-odds of data transformation characterizing data distribution. Patient numbers: h – healthy, d – diseased.

| Brain region | Expression change, fold to control | | Geo2R statistics | | | | | | | | | | GEO ID  (GSE) |
| --- | --- | --- | --- | --- | --- | --- | --- | --- | --- | --- | --- | --- | --- |
|  |  |  | *LYPD6* level | | | | *LYPD6B* level | | | | Patient number | |  |
|  | *LYPD6* | *LYPD6B* | LFC | *p* | t | B | LFC | *p* | t | B | h | d |  |
| **Alzheimer disease** | | | | | | | | | | | | | |
| Frontal cortex | 0.99 | n/a | -0.01 | 0.11 | 1.6 | -6.2 | n/a |  |  |  | 157 | 157 | 44771 |
| Visual cortex | 0.98 | n/a | -0.03 | **0.05** | 2 | -5.4 | n/a |  |  |  | 157 | 310 | 33000 |
| Olfactory bulbs | 1.08 | n/a | 0.11 | 0.47 | 0.2 | -0.7 | n/a |  |  |  | 20 | 19 | 113524 |
| **Autism** | | | | | | | | | | | | | |
| Dorsolateral prefrontal cortex | 1.08 | 1.21 | 0.11 | 0.28 | 0.1 | -1 | 0.28 | **0.03** | 0.1 | -2.1 | 39 | 13 | 102741 |
| Cerebellum | 0.95 | 0.93 | -0.07 | 0.16 | 1.5 | -5.1 | -0.1 | **0.03** | 2.4 | -3.6 | 12 | 14 | 38322 |
| Occipital lobe | 0.85 | 0.82 | -0.23 | 0.06 | 2.1 | -3.8 | -0.29 | 0.08 | 1.9 | -4 | 6 | 4 | 38322 |
| Total brain | 1.52 | 1.56 | 0.6 | 0.24 | -1 | -4.8 | 0.64 | 0.15 | -1 | -4.5 | 61 | 52 | 28475 |
| **Bipolar disorder** | | | | | | | | | | | | | |
| Dorsolateral prefrontal cortex | 0.94 | 0.84 | -0.09 | 0.50 | 0.1 | 0.8 | -0.26 | **0.03** | 0.1 | 2.2 | 24 | 24 | 80655 |
| Hippocampus | 0.98 | 0.91 | -0.02 | 0.80 | 0.3 | -5.2 | -0.14 | 0.18 | 1.4 | -4.6 | 18 | 17 | 53987 |
| Associative striatum | 1.02 | 1.02 | 0.03 | 0.73 | -0.4 | -5.3 | 0.03 | 0.68 | -0.4 | -5.3 | 18 | 17 | 53987 |
| Anterior cingulate cortex | 0.89 | 0.86 | -0.17 | 0.13 | 0.1 | 1.5 | -0.21 | 0.11 | 0.1 | 1.6 | 24 | 24 | 80655 |
| Nucleus accumbens | 0.88 | 0.75 | -0.18 | 0.20 | 0.2 | 1.3 | -0.42 | 0.14 | 0.3 | 1.5 | 22 | 22 | 80655 |
| **Chronic alcoholism** | | | | | | | | | | | | | |
| Hippocampus | 0.95 | 1.07 | -0.07 | 0.38 | 0.89 | -5.3 | 0.1 | 0.48 | -0.7 | -5.4 | 19 | 20 | 44456 |
| **Eating disorders** | | | | | | | | | | | | | |
| Dorsolateral prefrontal cortex | 0.93 | 1.00 | -0.11 | 0.06 | 1.9 | -5.3 | 0 | 0.97 | 0.04 | -7 | 102 | 15 | 60190 |
| **Epilepsy** | | | | | | | | | | | | | |
| Frontal cortex | 1.01 | 1.09 | 0.02 | 0.94 | 0.2 | -0.1 | 0.13 | 0.6 | 0.2 | -0.5 | 12 | 24 | 186334 |
| Hippocampus | 1.08 | 0.83 | 0.11 | 0.73 | 0.3 | -0.4 | -0.27 | 0.34 | 0.3 | 1 | 10 | 10 | 186334 |
| **Epilepsy (with hippocampal sclerosis)** | | | | | | | | | | | | | |
| Frontal cortex | 1.01 | 1.02 | 0.01 | 0.96 | 0.2 | -0.1 | 0.03 | 0.92 | 0.3 | -0.1 | 12 | 12 | 186334 |
| **Huntington disease** | | | | | | | | | | | | | |
| Frontal cortex | 1.02 | n/a | 0.03 | **0.0005** | -3.5 | -1.4 | n/a |  |  |  | 157 | 157 | 33000 |
| Motor cortex | 1.09 | 1.16 | 0.13 | 0.3 | 0.1 | -1 | 0.22 | 0.13 | 0.2 | -1.5 | 7 | 7 | 79666 |
| **Major depressive disorder** | | | | | | | | | | | | | |
| Dorsolateral prefrontal cortex | 0.96 | 0.95 | 0.05 | 0.58 | 0.09 | 0.56 | 0.07 | 0.45 | 0.1 | 0.8 | 24 | 23 | 80655 |
| Hippocampus | 0.97 | 0.96 | -0.06 | 0.56 | 0.6 | -5 | -0.18 | 0.16 | 1.4 | -4.5 | 18 | 17 | 53987 |
| Amygdala | 0.96 | 0.88 | -0.09 | 0.16 | 1.5 | -4.6 | 0.05 | 0.32 | -1 | -5.1 | 14 | 14 | 53987 |
| Associative striatum | 0.94 | 1.03 | -0.09 | 0.36 | 0.9 | -5 | -0.05 | 0.38 | 0.9 | -5 | 18 | 16 | 53987 |
| Anterior cingulate cortex | 0.94 | 0.97 | -0.06 | 0.52 | 0.1 | 0.6 | 0.06 | 0.57 | 0.1 | -0.6 | 24 | 24 | 80655 |
| Nucleus accumbens | 0.96 | 1.04 | -0.18 | 0.20 | 0.1 | 1.3 | -0.42 | 0.14 | 0.3 | 1.5 | 22 | 22 | 80655 |
| **Multiple sclerosis** | | | | | | | | | | | | | |
| Frontal cortex | 0.79 | 0.72 | -0.26 | 0.74 | 0.8 | 0.3 | -0.47 | 0.3 | 0.5 | 0.99 | 5 | 5 | 123496 |
| Hippocampus | 0.48 | 0.71 | -0.17 | 0.29 | 0.2 | 1 | -0.49 | 0.1 | 0.3 | 1.66 | 5 | 5 | 123496 |
| **Normal ageing** | | | | | | | | | | | | | |
| Frontal cortex | 0.65 | 0.90 | -0.63 | **0.01** | 3 | -2.4 | -0.15 | 0.6 | 0.6 | -6 | 9 | 18 | 48350 |
| Hippocampus | 0.75 | 0.86 | -0.41 | **0.02** | 2.6 | -3.5 | -0.22 | 0.1 | 1.9 | -4.8 | 9 | 18 | 48350 |
| **Obsessive-compulsive disorder** | | | | | | | | | | | | | |
| Dorsolateral prefrontal cortex | 0.97 | 1.05 | -0.05 | 0.39 | 0.9 | -6.7 | 0.07 | **0.02** | -2.3 | -4.4 | 102 | 16 | 60190 |
| **Parkinson disease** | | | | | | | | | | | | | |
| Substantia nigra | 1.17 | 1.69 | 0.23 | 0.64 | -0.5 | -5.7 | 0.76 | 0.15 | -1.5 | -4.8 | 9 | 16 | 49036 |
| **Schizophrenia** | | | | | | | | | | | | | |
| Dorsolateral prefrontal cortex | 0.92 | 0.84 | -0.12 | 0.29 | 0.1 | 1 | -0.25 | **0.02** | 0.11 | 2.4 | 24 | 24 | 80655 |
| Hippocampus | 0.83 | 0.93 | -0.27 | **0.04** | 2.2 | -3.9 | -0.1 | 0.49 | 0.7 | -5.9 | 18 | 15 | 53987 |
| Associative striatum | 0.99 | 1.09 | -0.01 | 0.88 | 0.2 | -5.7 | 0.12 | 0.21 | -1.3 | -4.9 | 18 | 18 | 53987 |
| Anterior cingulate cortex | 0.87 | 0.93 | -0.2 | 0.06 | 0.1 | 1.9 | -0.1 | 0.3 | 0.1 | 1 | 24 | 24 | 80655 |
| Nucleus accumbens | 0.85 | 0.87 | -0.23 | 0.47 | 0.3 | 0.7 | -0.2 | 0.1 | 0.1 | 1.5 | 22 | 23 | 80655 |

## Supplementary Table 2. Schedule of behavior test battery.

| Testing day (day of administration) | Cognitive tests (test time)  Vehicle (n = 20), ws-Lypd6 (n = 19), ws-Lypd6b (n = 18) | | |
| --- | --- | --- | --- |
| 1 (15) | Open field test  (12:00 – 16:00) | Hypophagia: training (16:00 – 17:00) | Rotarod  (17:00 – 18:00) |
| 2 (16) | Elevated plus maze  (12:00 – 16:00) | Hypophagia: training (16:00 – 17:00) | Rotarod  (17:00 – 18:00) |
| 3 (17) |  | Hypophagia: training (16:00 – 17:00) | Rotarod  (17:00 – 18:00) |
| 4 (18) | Novel object recognition:  training  (11:00 – 12:00; 19:00 – 20:00) | Hypophagia: testing  (16:00 – 17:00) | Rotarod  (17:00 – 18:00) |
| 5 (19) | Novel object recognition:  training  (11:00 – 12:00; 19:00 – 20:00) | Novel odor recognition: training  (14:00 – 16:00) | Rotarod  (17:00 – 18:00) |
| 6 (20) | Novel object recognition:  training  (11:00 – 12:00; 19:00 – 20:00) | Novel odor recognition: training  (14:00 – 16:00) | Rotarod  (17:00 – 18:00) |
| 7 (21) | Novel object recognition: training (11:00 – 12:00) and testing (19:00 – 20:00) | Novel odor recognition: testing  (14:00 – 16:00) | Rotarod  (17:00 – 18:00) |

## Supplementary Table 3. Antibodies used in the study.

| Target | Manufacturer | Catalog No. | Host | RRID | Dilution | Incubation time, buffer, T˚C |
| --- | --- | --- | --- | --- | --- | --- |
| **Western blotting** | | | | | | |
| *Primary antibodies* | | | | | | |
| Lypd6 | Antibodies online, Aachen, Germany | ABIN5582866 | rabbit | AB_3076434 | 1:1000 | overnight, 5% skim milk/TBST, 4˚C |
| Lypd6b | Abcam, Cambridge, UK | ab201163 |  | AB_3076435 |  |  |
| α3 nAChR | Antibodies online | ABIN1867228 |  | AB_3076415 |  |  |
| α4 nAChR |  | ABIN5013334 |  | AB_3076437 |  |  |
| α7 nAChR | Abcam | ab10096 |  | AB_296838 |  |  |
| β2 nAChR | Antibodies online | ABIN5611311 | mouse | AB_3076439 |  |  |
| β-actin | R&D Systems, Minneapolis, USA | MAB8929 |  | AB_3076436 | 1:2000 | 1 h, 5% skim milk/TBST,  Room temp. |
| GAPDH | ServiceBio, Wuhan, Hubei, China | GB15004-100 | rabbit | AB_2943040 |  |  |
| *Secondary antibodies* | | | | | | |
| HRP anti-rabbit | Jackson Immunoresearch, West Grove, USA | 111-035-003 | goat | AB_2313567 | 1:5000 | 1 h, 1% skim milk/TBST,  Room temp. |
| HRP anti-mouse |  | 715-005-150 | donkey | AB_2340758 |  |  |
| **Affinity purification** | | | | | | |
| *Primary antibodies* | | | | | | |
| α3 nAChR | Antibodies online | ABIN1867228 | rabbit | AB_3076415 | 1:1000 | overnight, 5% skim milk/TBST, 4˚C |
| α4 nAChR | Abcam | ABIN5013334 |  | AB_3076437 |  |  |
| α6 nAChR | Antibodies online | ABIN5611358 | mouse | AB_3076438 |  |  |
| α7 nAChR | Abcam | ab10096 | rabbit | AB_296838 |  |  |
| β2 nAChR | Antibodies online | ABIN5611311 | mouse | AB_3076439 |  |  |
| α5 GABA_A_ | Novus Biologicals | NBP2-45752 | rabbit | AB_3076440 |  |  |
| Cytochrome C | Abcam | ab13575 | mouse | AB_300470 |  |  |
| *Secondary antibodies* | | | | | | |
| HRP anti-rabbit | Jackson Immunoresearch | 111-035-003 | goat | AB_2313567 | 1:5000 | 1 h, 1% skim milk/TBST,  Room temp |
| HRP anti-mouse |  | 715-005-150 | donkey | AB_2340758 |  |  |
| **Co-localization analysis (sequential staining, first – Lypd6b, then nAChRs)** | | | | | | |
| *Primary antibody* | | | | | | |
| Lypd6b | Abcam, Cambridge, UK | ab201163 |  | AB_3076435 | 1:200 | overnight, PBS, 4˚C |
| *Secondary antibody* | | | | | | |
| Alexa647-  anti-rabbit | Jackson  Immunoresearch | 111-605-003 | goat | AB_2338072 | 1:1000 | 1 h, PBS,  Room temp |
| *Primary antibodies (after staining endogenous Lypd6b)* | | | | | | |
| α3 nAChR | Antibodies online | ABIN1867228 | rabbit | AB_3076415 | 1:200 | 3 h, PBS,  Room temp |
| α4 nAChR |  | ABIN5013334 |  | AB_3076437 |  |  |
| α7 nAChR | Abcam | ab10096 |  | AB_296838 |  |  |
| β2 nAChR | Antibodies online | ABIN5611311 | mouse | AB_3076439 |  |  |
| *Secondary antibodies* | | | | | | |
| TRITC- anti-rabbit | Jackson  Immunoresearch | 711-025-152 | donkey | AB_2340588 | 1:1000 | 1 h, PBS,  Room temp |
| TRITC- anti-mouse |  | 715-025-151 | donkey | AB_2340767 |  |  |

## Supplementary Table 4. Primers used for qPCR.

| Gene | Primer | | Amplicon size |
| --- | --- | --- | --- |
|  | Forward | Reverse |  |
| *β-actin* | GCAGCCACTGTCGAGTC | ACGATGGAGGGGAATACAGC | 190 |
| *Sdha* | AACACTGGAGGAAGCACACC | AGTAGGAGCGGATAGCAGGA | 135 |
| *Rpl13a* | CGCTGTGAAGGCATCAACAT | TGGCCTCTCTTGGTCTTGTG | 104 |
| *Lrp6* | AAAACCGCACCATCATCCAG | AGGCATCCACACCAAAAAC | 161 |
| *Lypd6* | CTGTCACCAAACGCTGTGTC | TTGTAGCCTTCGTGCTCTGAG | 97 |
| *Lypd6b* | ACTTGCGAAAATGCA GGGGAT | GCTGGTGAAGTGATGAACTGTCA | 76 |
| *Chrna7* | TGGTCCTATGGAGGGTGGTC | TAGAGTGTCCTACGGCGCAT | 185 |
| *Lynx1* | CAACACCGCACGAAGTGTG | GCCTGAGCTCTTGGTCTCTT | 101 |
| *Actn1* | GACTTCCGAGATGGCCTGAA | ACACCAGCTTGACCCCTTTG | 154 |
| *Fos* | ACGGAGAATCCGAAGGGAAC | CGGTGGGCTGCCAAAATAAA | 186 |
| *Jun* | GCGCACGCTCCTAAACAAAC | AGAACGGTCCGTCACTTCAC | 135 |
| *Gfap* | AAAGCCTCAAGGAGGAGATGG | TGCTTTTGCCCCCTCGGAT | 177 |
| *Dlg4* | ATGCTCCCCCAGACATCACA | TCACGATCCGCCTTGGTTC | 183 |
| *Syn1* | TGCCAATGGTGGATTCTCCG | CAGCCCAATGACCAAACTGC | 148 |
| *Syp* | TGCCAACAAGACGGAGAGTG | GAATTCAGCCGACGAGGAGT | 151 |
| *Snca* | TTGGGGAAAACAGGAAGAATCGG | CTGTTGTCACTCCATGAACCAC | 237 |
| *Tubb* | AGTAAACCGTAGCCATGAGGG | GTGGGGTCGATGCCATGTTC | 112 |
| *Tmem35a* | GAGGGATTAGTTGGGACCTGC | GGCTCGAACGTAGCTCTTGTA | 183 |


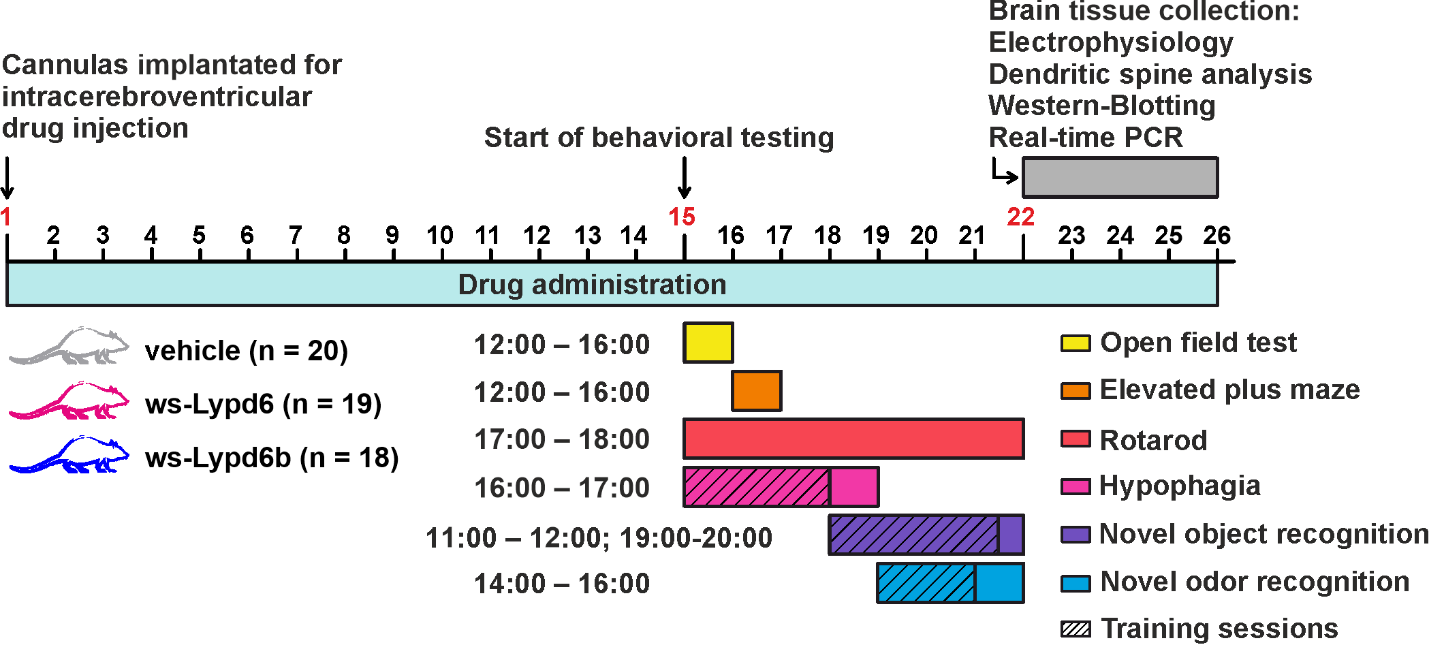


Supplementary Figure 1. **Experimental design.**


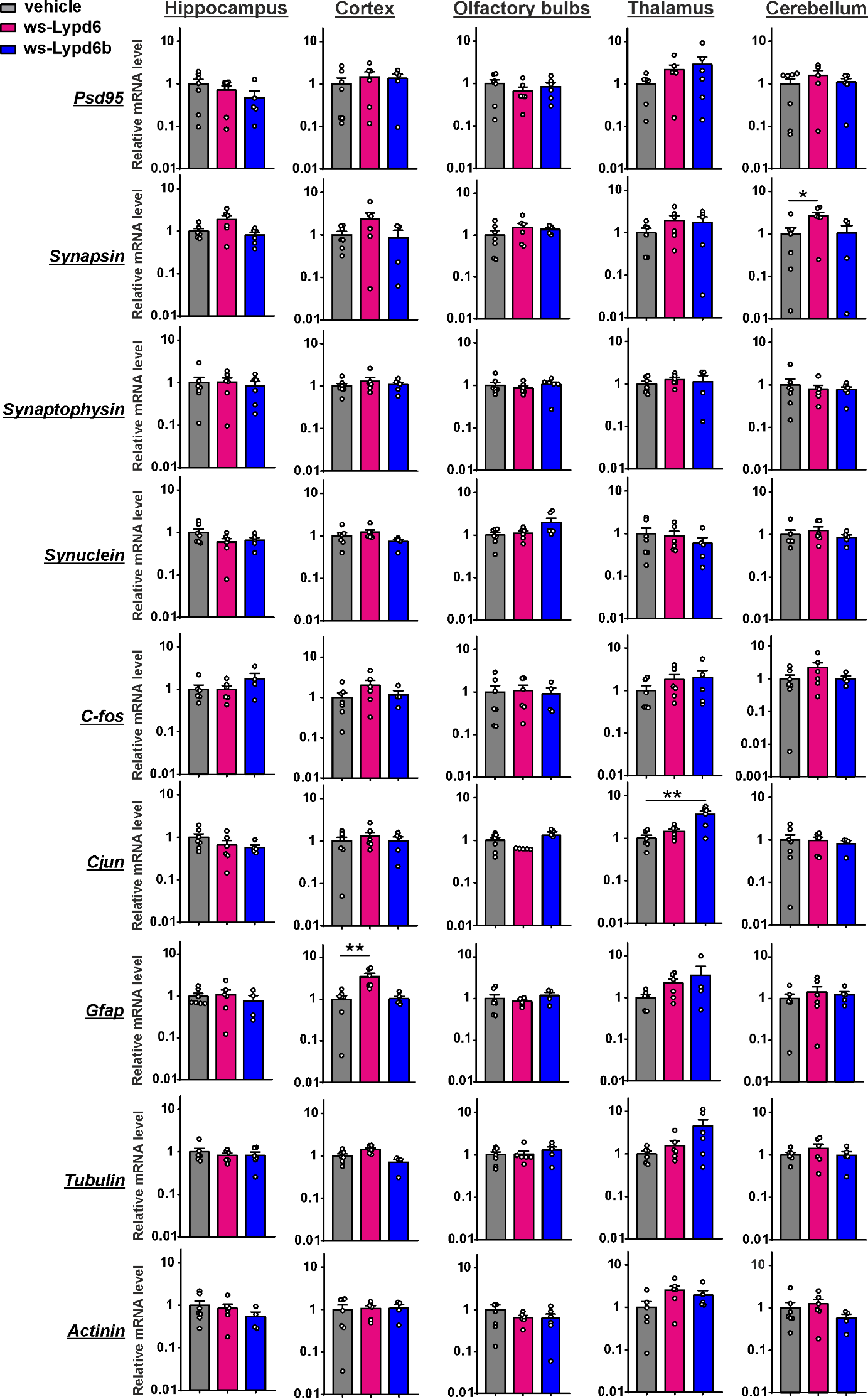


Supplementary Figure 2. **Gene expression** **of synaptic factors in the brain.**

Expression level of genes *Dlg4*, *Syn1*, *Syp*, *Snca*, *Fos*, *Jun*, *Gfap*, *Tubb*, *Actn1*, coding Psd-95, Synapsin-1, Synaptophysin, Alpha-Synuclein, C-fos, C-jun, Gfap, Tubulin Beta Class I, Alpha-Actinin-1 proteins, respectively, in the hippocampus, cortex, olfactory bulbs, thalamus, and cerebellum, respectively. Data are presented as the ratio of expression level of the studied genes in experimental groups to the expression level in control (vehicle) group ± SEM (n = 4 – 7). * (p < 0.05) and ** (p < 0.01) indicate significant difference from vehicle group according to one-way ANOVA test followed by *post hoc* Dunnet test.


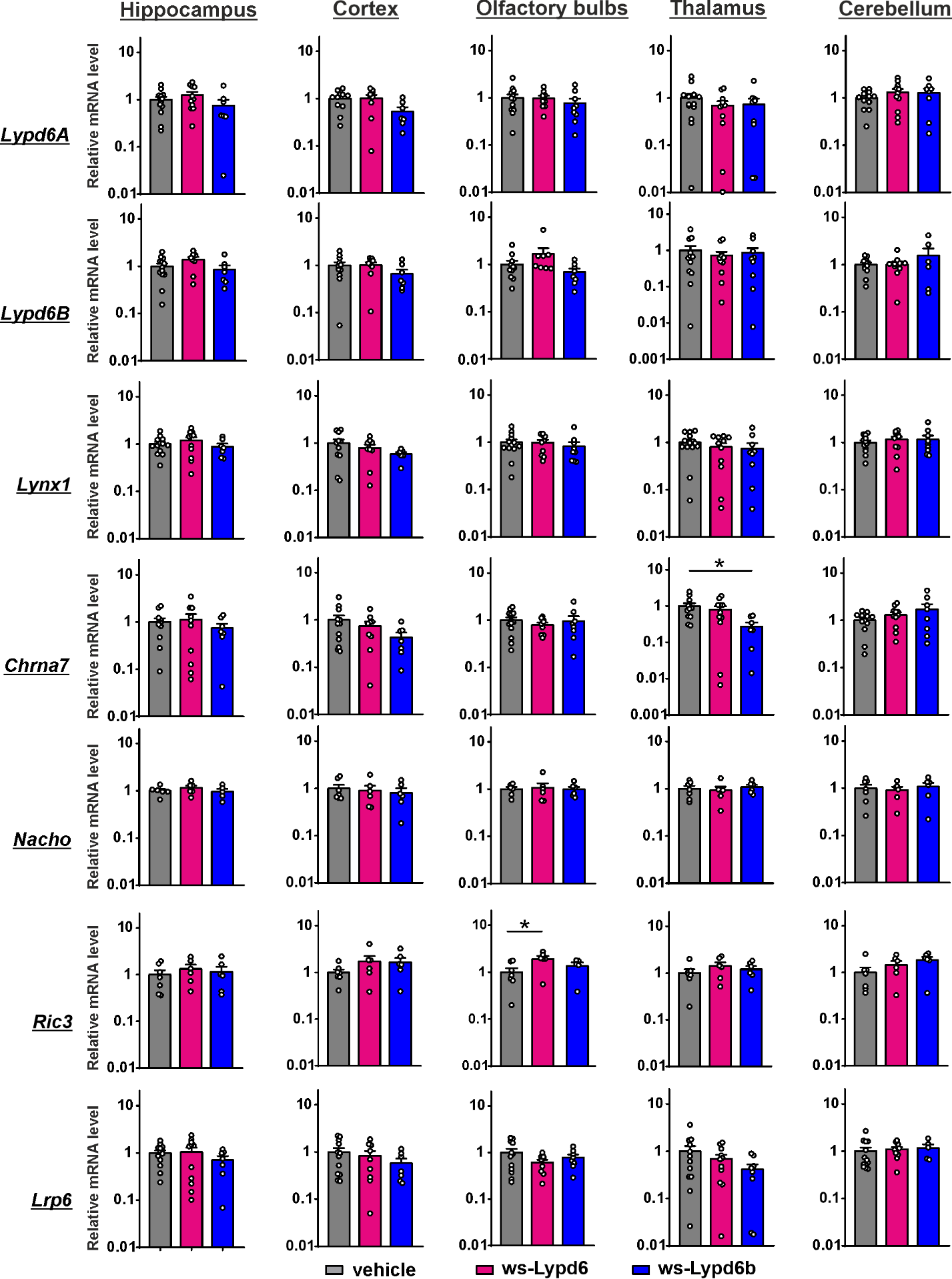
 Supplementary Figure 3. **Expression of the genes coding Lypd6, Lypd6b, Lynx1, α7-nAChR subunit, and the chaperones of α7-nAChR (NACHO and RIC3) in the cortex, hippocampus, olfactory bulbs, thalamus, and cerebellum.**

Genes *Lypd6, Lypd6b, Chrna7, Lynx1, Tmem35a and Ric3.* Data are presented as the ratio of expression level of the studied genes in experimental groups to the expression level in control (vehicle) group ± SEM (n = 4 – 13). * (p < 0.05) indicates difference from vehicle group according to one-way ANOVA test followed by Dunnet’s post hoc test.


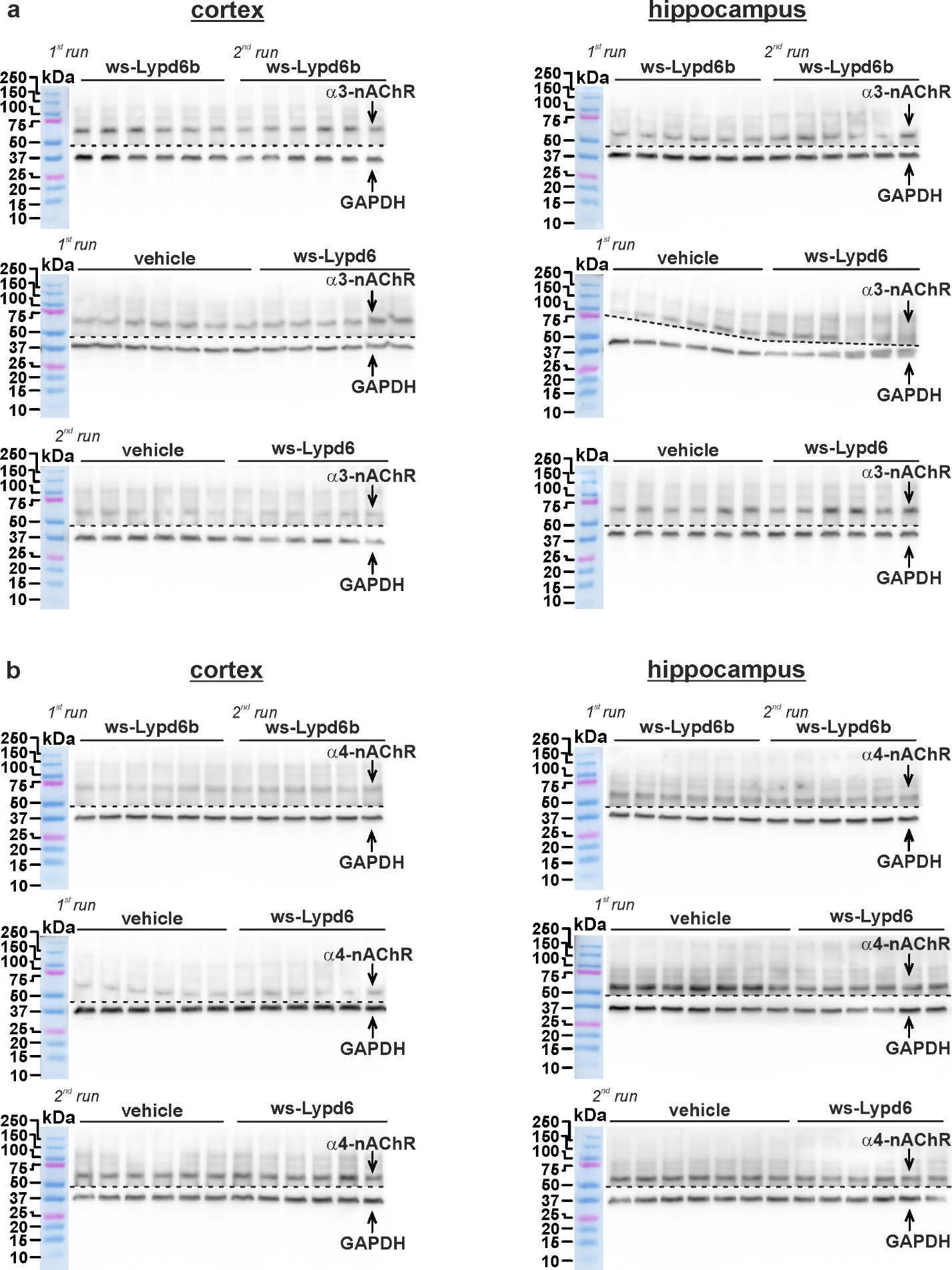


Supplementary Figure 4. **Whole Western-blotting membranes for analysis of the expression of α3 (a) and α4 (b) nAChR subunits in the hippocampus and cortex.**

Staining of β-actin and GAPDH was performed on the same membranes as staining of α3 and α4 nAChR subunits, the cut line id marked by dashed line.


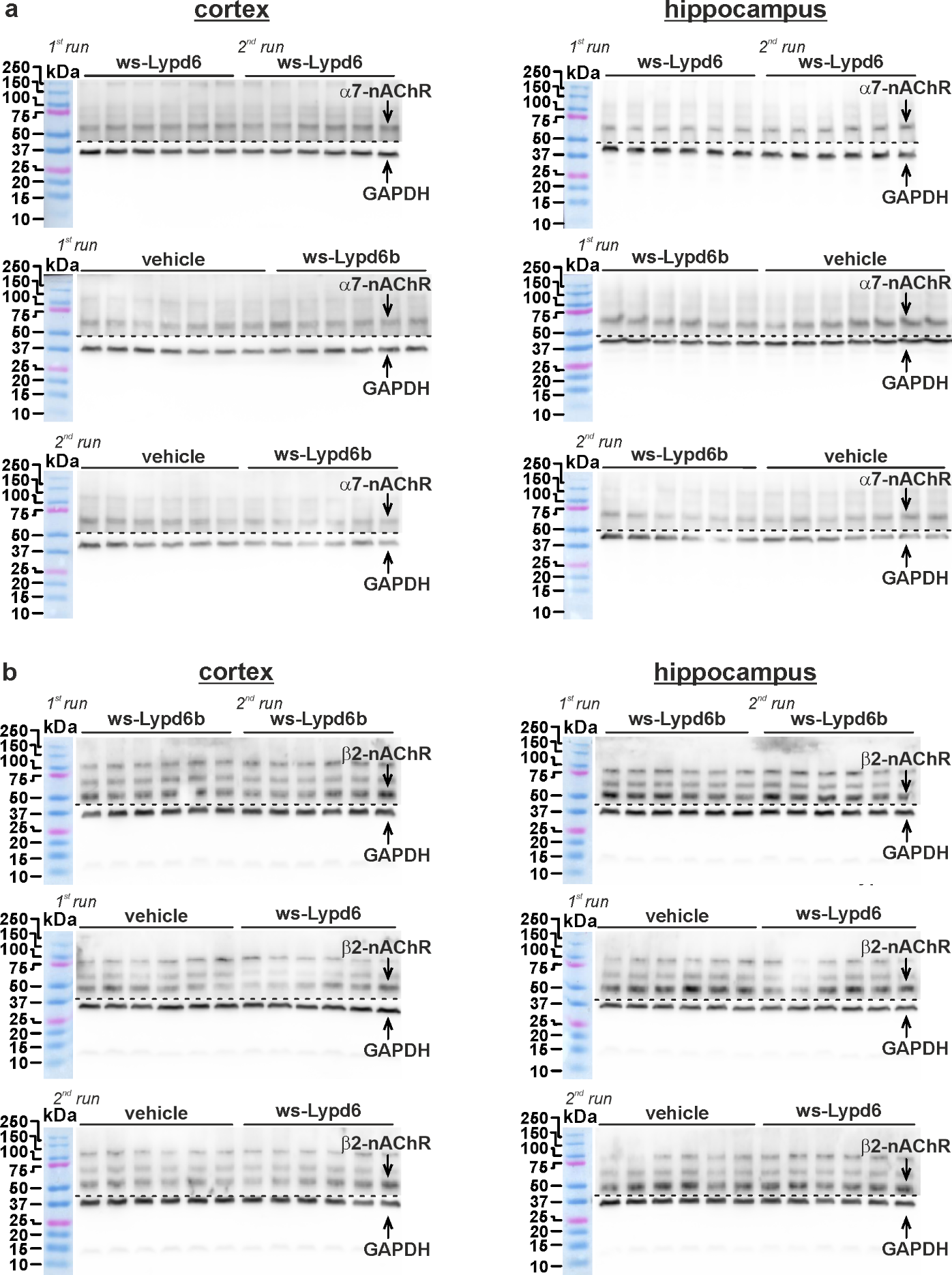


Supplementary Figure 5. **Whole Western-blotting membranes for analysis of expression of α7 (a) and β2 (b) nAChR subunits in the hippocampus and cortex.**

Staining of β-actin and GAPDH was performed on the same membranes as staining of α7 and β2 nAChR subunits, the cut line is marked by dashed line.


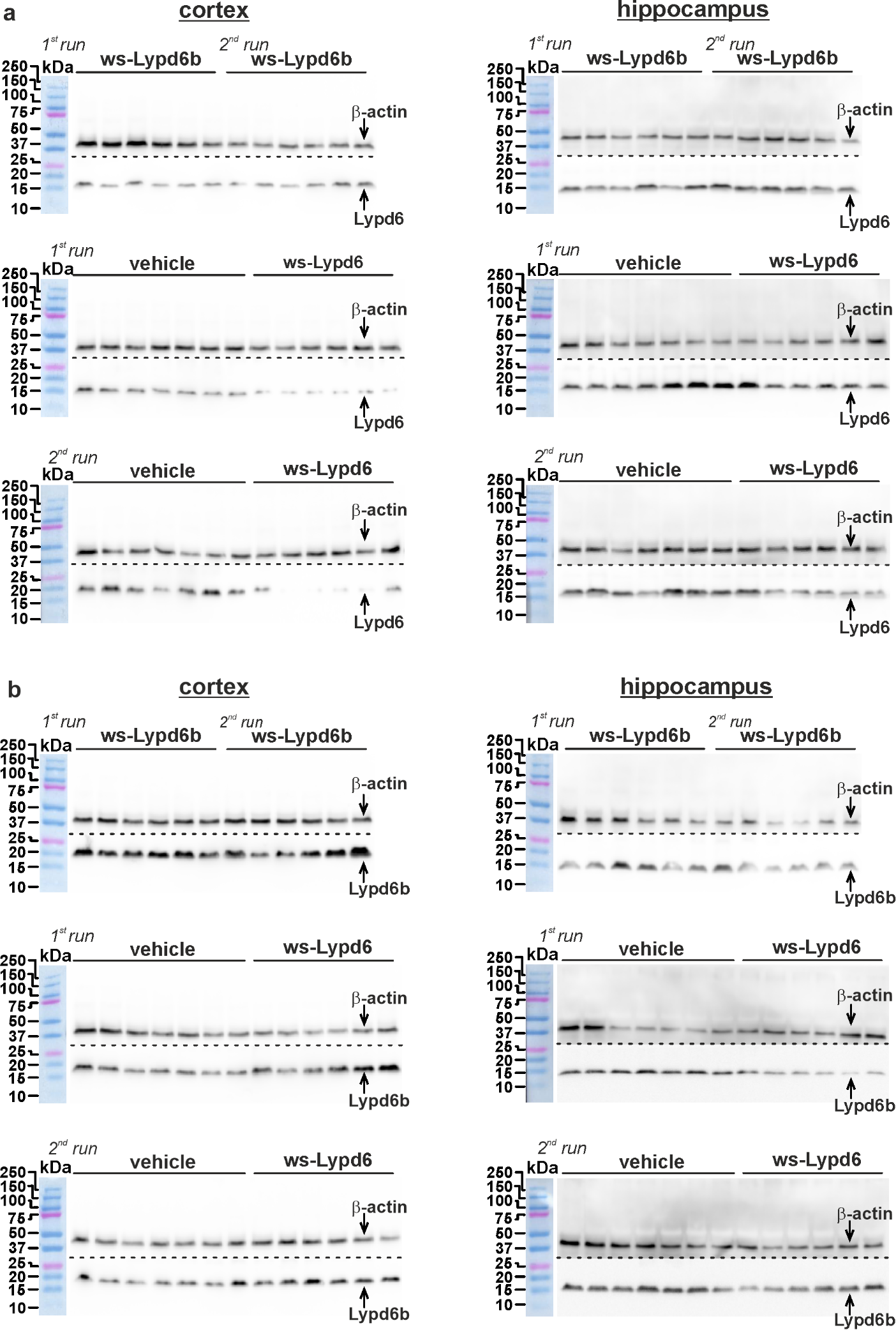


Supplementary Figure 6. **Whole Western-blotting membranes for analysis of expression of** **Lypd6 (a) and Lypd6b (b) in the hippocampus and cortex.**

Staining of β-actin was performed on the same membranes as staining of Lypd6 and Lypd6b, the cut line is marked by dashed line.


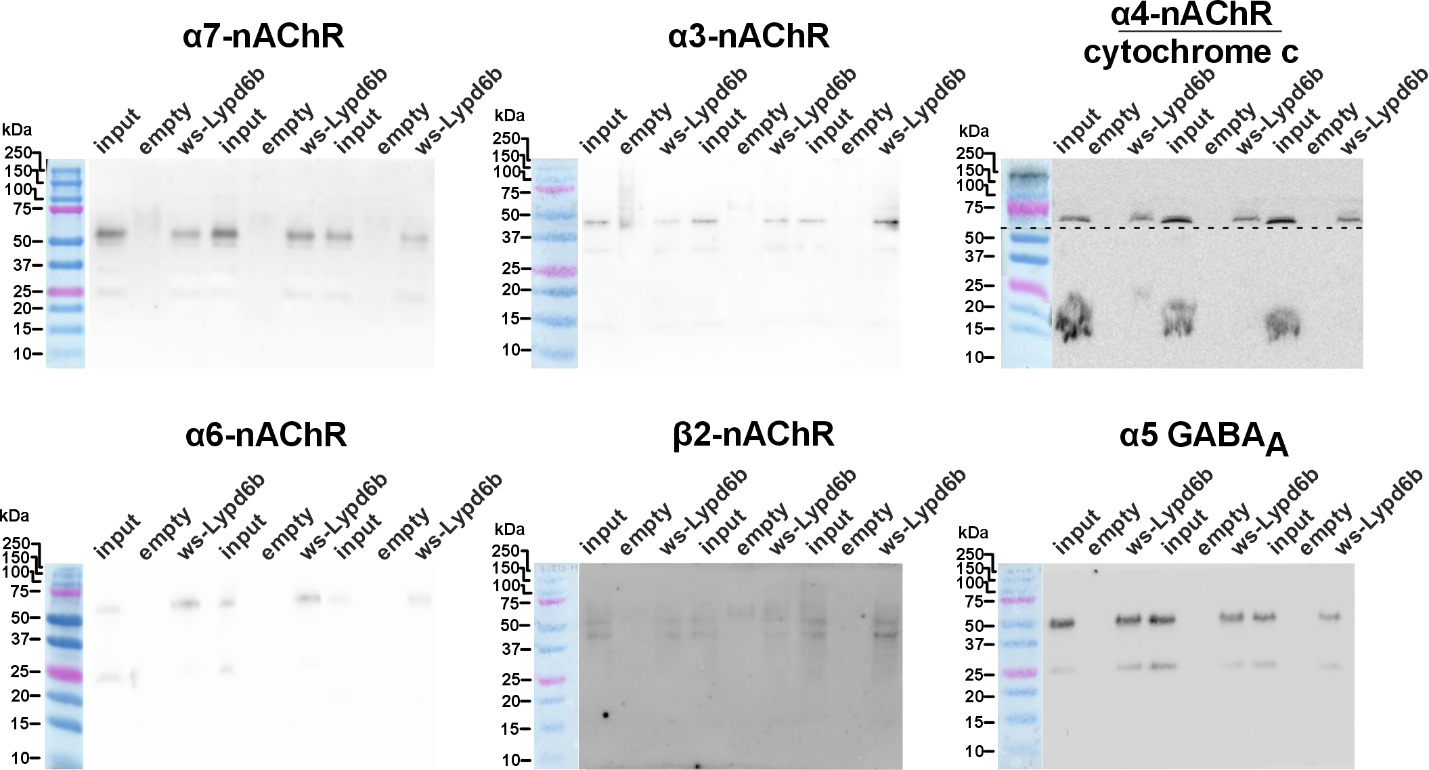


Supplementary Figure 7. **Whole Western-blotting membrane for analysis of interactome of ws-Lypd6b.**

Staining of α4 nAChR subunit and cytochrome C was performed on the same membrane, the cut line is marked by dashed line.


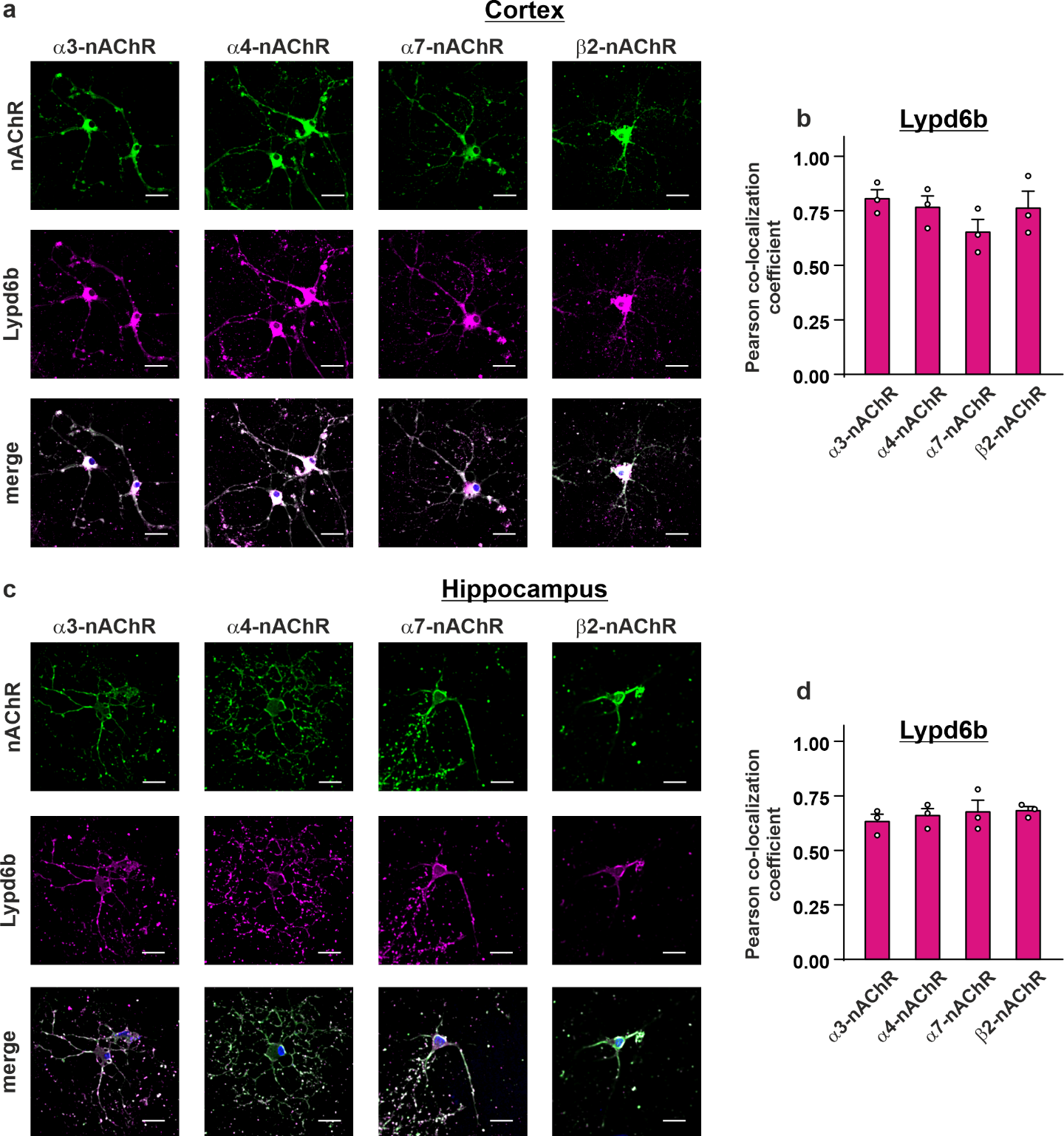


Supplementary Figure 8. **Co-localization of endogenous Lypd6b with different nAChR subunits in the cortical and hippocampal neurons.**

**a,c.** Neurons from the cortex **(a)** and hippocampus **(c)** were sequentially stained by anti-Lypd6b antibody and Alexa647 conjugated secondary Abs, and after that by antibodies to α7, α3, α4, α6, and β2 nAChR subunits and TRITC-labelled secondary antibodies. **b,d.** Pearson co-localization coefficients for Lypd6b stained in the cortex **(b)** and hippocampus **(d)** with different nAChR subunits (n = 3 ± SEM). Scale = 25 µm.
