# Supplementary material for: Upregulation of cholinergic modulators Lypd6 and Lypd6b associated with autism drives anxiety and cognitive decline
Source: Cell Death Discov. 2024 Oct 21;10:444. doi: 10.1038/s41420-024-02211-z (PMC11494011; doi:10.1038/s41420-024-02211-z)

Original Western blots

Up-regulation of cholinergic modulators Lypd6 and Lypd6b associated with autism drives anxiety and cognitive decline

Running title: Lypd6 and Lypd6b drive cognitive decline

Aizek B. Isaev[2,3†], Maxim L. Bychkov[2†], Dmitrii S. Kulbatskii[2], Alexander A. Andreev-Andrievskiy[4,5], Mikhail A. Mashkin[5], Mikhail A. Shulepko[1], Olga V. Shlepova[2,3], Eugene V. Loktyushov[6], Alexander V. Latanov[4], Mikhail P. Kirpichnikov[2,4], Ekaterina N. Lyukmanova[1,2,3,4]

[1]Shenzhen MSU-BIT University, 518172, Shenzhen, China

[2]Shemyakin-Ovchinnikov Institute of Bioorganic Chemistry, Russian Academy of Sciences, 119997, Moscow, Russia.

[3]Moscow Center for Advanced Studies, Moscow, Russia

[4]Interdisciplinary Scientific and Educational School of Moscow University «Molecular Technologies of the Living Systems and Synthetic Biology», Faculty of Biology, Lomonosov Moscow State University, 119234, Moscow, Russia.

[5]Institute for biomedical problems of Russian Academy of Sciences, 123007, Moscow, Russia

[6]Pushchino Scientific Center for Biological Research of the Russian Academy of Sciences, Institute for Biological Instrumentation, 142290, Pushchino, Russia *Correspondence: lyukmanova_ekaterina@smbu.edu.cn (E.N.L.)

† These authors contributed equally to this work

Original Western blots

Original images with marked areas for Figure 5 and Supplementary Figures 4 and 5:

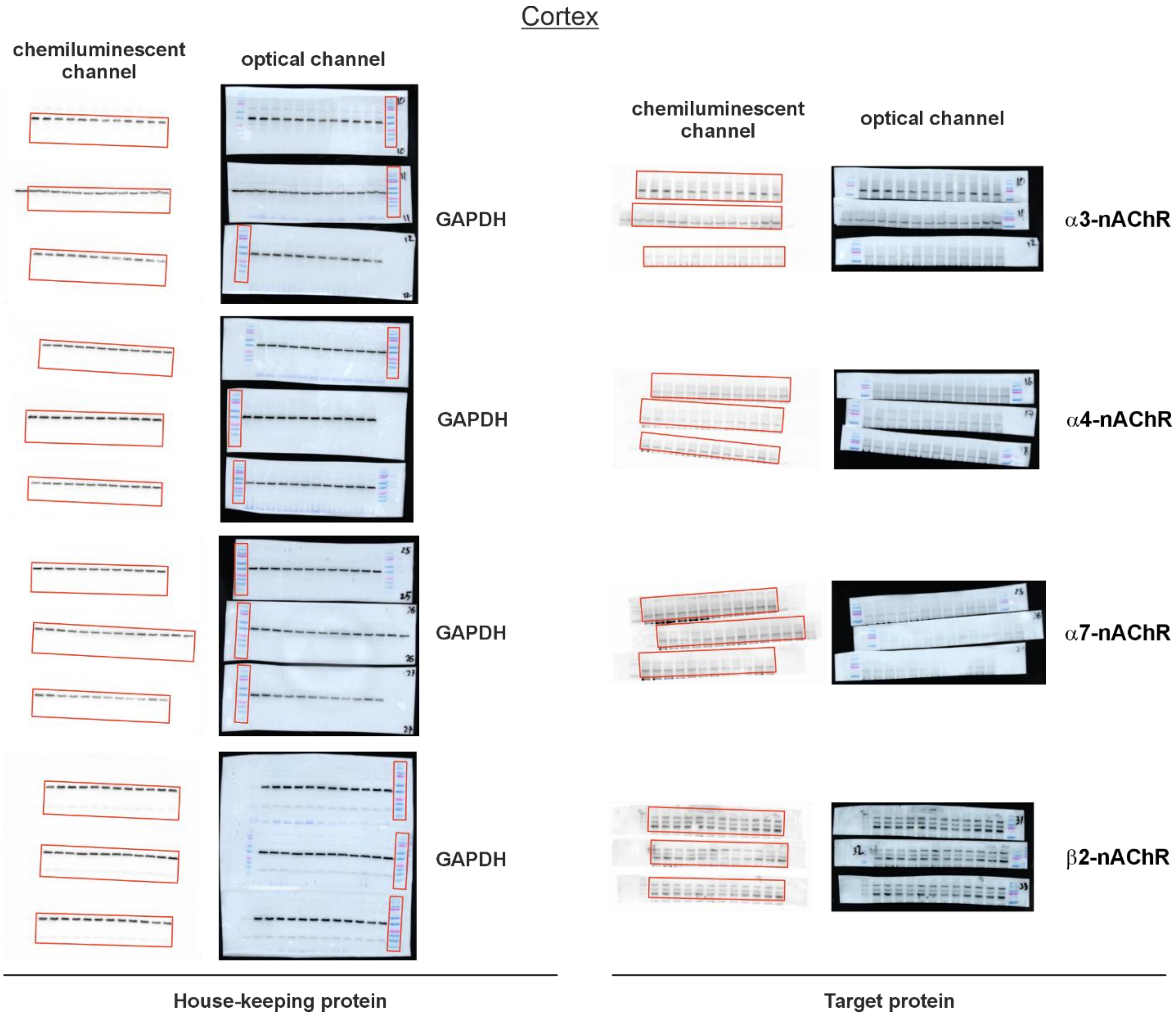

Original Western blots

Original images with marked areas for Figure 5 and Supplementary Figures 4 and 5:

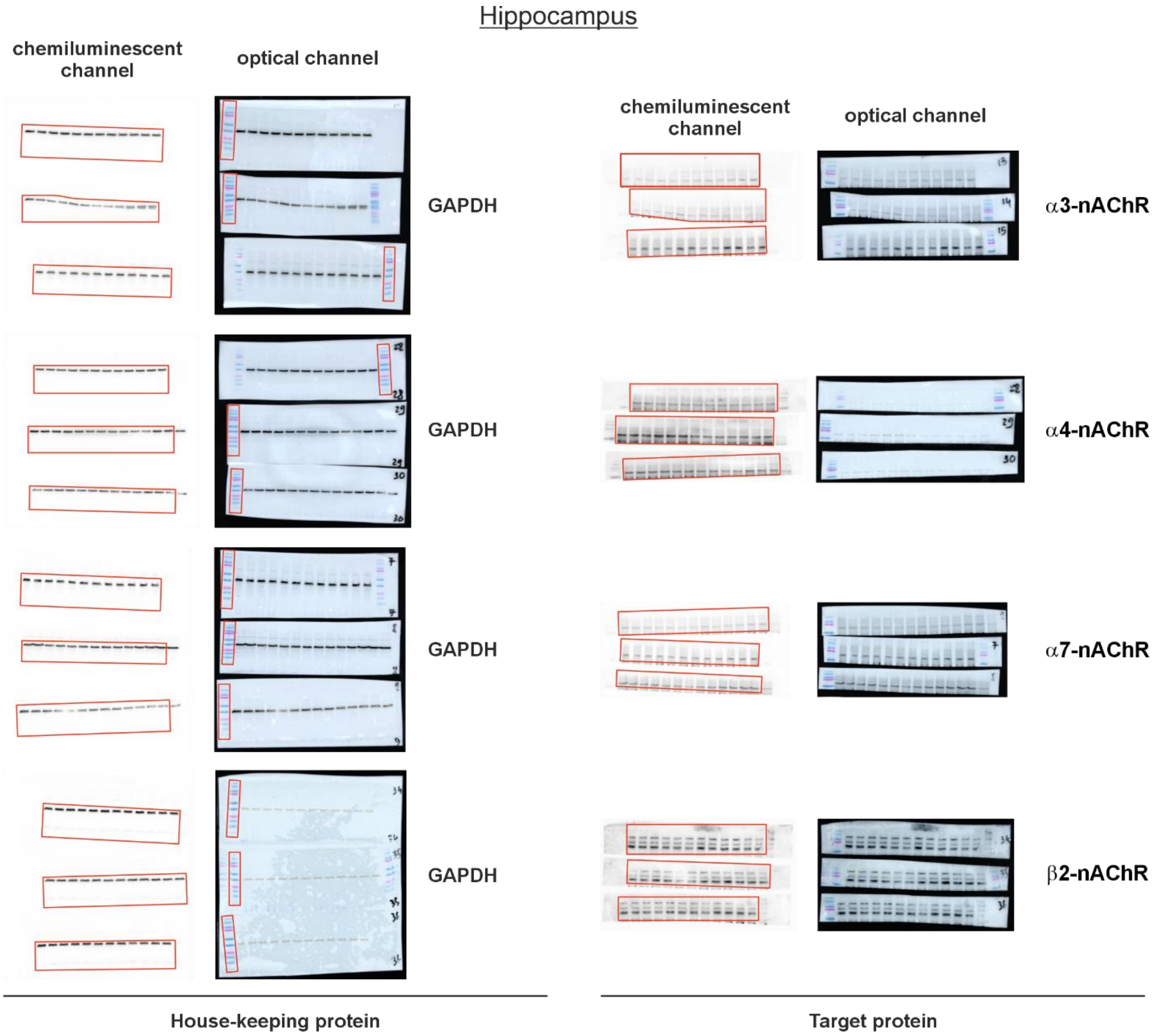

Original Western blots

Original images with marked areas for Figure 6 and Supplementary Figure 6:

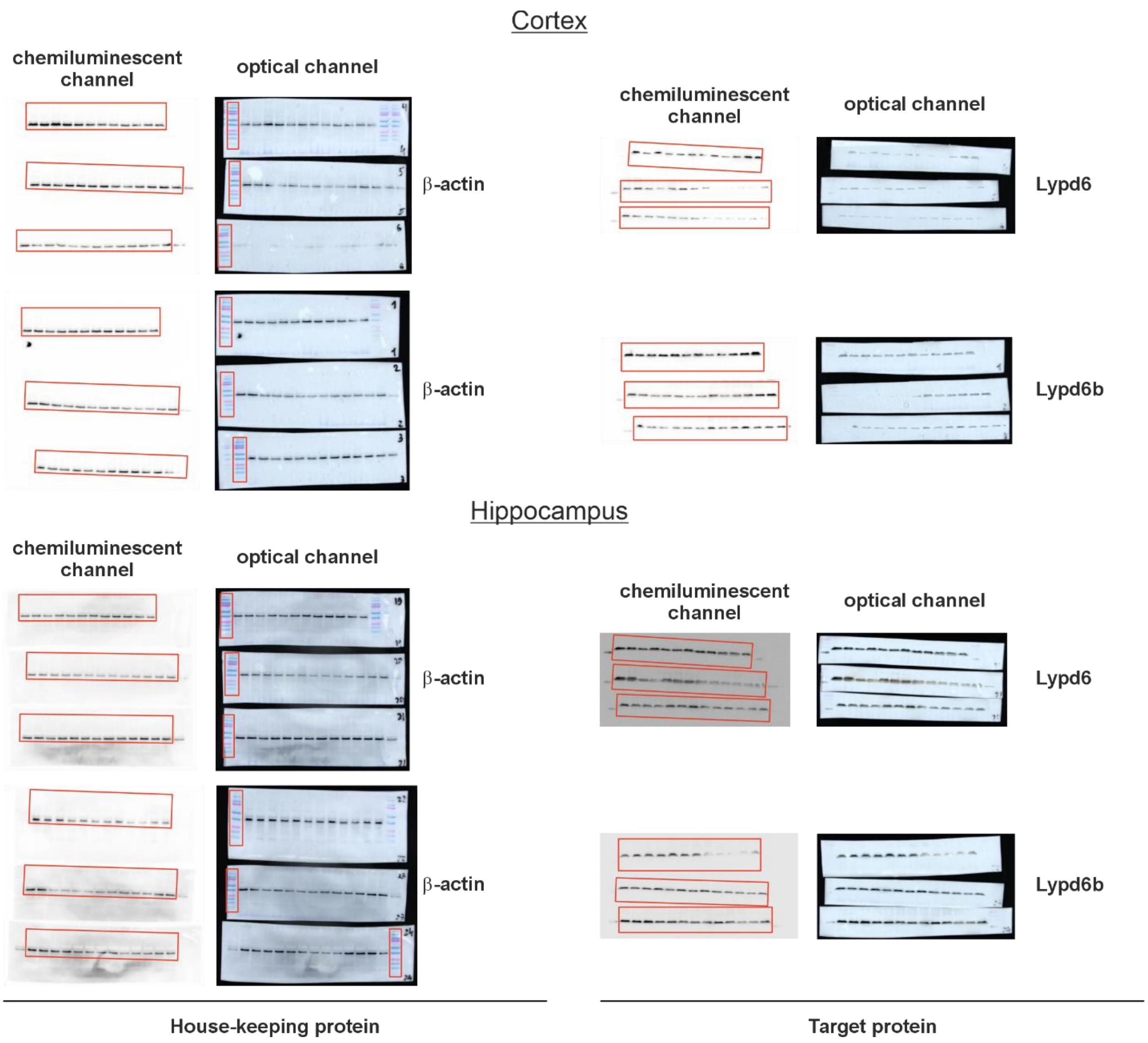

Original Western blots

Original images with marked areas for Figure 7 and Supplementary Figure 7:

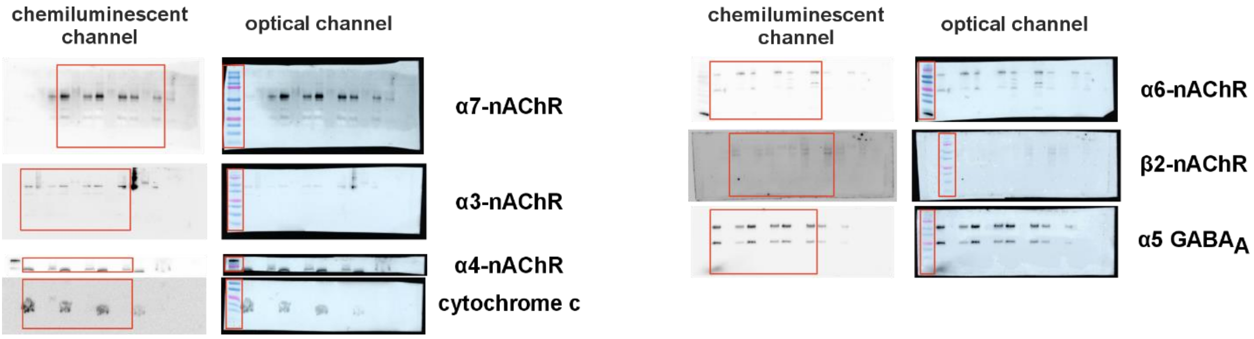

Supplement: Supplementary file 2 — Original WB images [file 41420_2024_2211_MOESM2_ESM.pdf]
